# *VEGF-A*, *VEGFR1* and *VEGFR2* single nucleotide polymorphisms and outcomes from the AGITG MAX trial of capecitabine, bevacizumab and mitomycin C in metastatic colorectal cancer

**DOI:** 10.1038/s41598-021-03952-y

**Published:** 2022-01-24

**Authors:** Fiona Chionh, Val Gebski, Sheren J. Al-Obaidi, Jennifer K. Mooi, Maressa A. Bruhn, Chee K. Lee, Anderly C. Chüeh, David S. Williams, Andrew J. Weickhardt, Kate Wilson, Andrew M. Scott, John Simes, Jennifer E. Hardingham, Timothy J. Price, John M. Mariadason, Niall C. Tebbutt

**Affiliations:** 1 Olivia Newton-John Cancer Research Institute, Heidelberg, VIC 3084 Australia; 2 NHMRC Clinical Trials Centre, University of Sydney, Sydney, NSW 2006 Australia; 3 Haematology-Oncology Department, Basil Hetzel Institute, The Queen Elizabeth Hospital, Woodville, SA 5001 Australia; 4 Department of Medicine, Austin Health, The University of Melbourne, Melbourne, VIC 3084 Australia; 5 Cancer Program and Department of Biochemistry and Molecular Biology, Biomedicine Discovery Institute, Monash University, Clayton, VIC 3168 Australia; 6 School of Cancer Medicine, La Trobe University, Bundoora, VIC 3086 Australia; 7 Department of Pathology, Austin Health, Heidelberg, VIC 3084 Australia; 8 Department of Clinical Pathology, The University of Melbourne, Parkville, VIC 3010 Australia; 9 Department of Molecular Imaging and Therapy, Austin Health, Heidelberg, VIC 3084 Australia; 10 School of Medicine, University of Adelaide, Adelaide, SA 5005 Australia; 11 Department of Medical Oncology, Austin Health, Heidelberg, VIC 3084 Australia; 12 Department of Surgery, The University of Melbourne, Parkville, VIC 3010 Australia

**Keywords:** Oncology, Genetic markers, Gastrointestinal cancer, Colorectal cancer, Tumour biomarkers, Predictive markers, Prognostic markers, Translational research, Targeted therapies, Cancer, Tumour angiogenesis

## Abstract

The phase III MAX clinical trial randomised patients with metastatic colorectal cancer (mCRC) to receive first-line capecitabine chemotherapy alone or in combination with the anti-VEGF-A antibody bevacizumab (± mitomycin C). We utilised this cohort to examine whether single nucleotide polymorphisms (SNPs) in *VEGF-A*, *VEGFR1*, and *VEGFR2* are predictive of efficacy outcomes with bevacizumab or the development of hypertension. Genomic DNA extracted from archival FFPE tissue for 325 patients (69% of the MAX trial population) was used to genotype 16 candidate SNPs in *VEGF-A*, *VEGFR1*, and *VEGFR2,* which were analysed for associations with efficacy outcomes and hypertension. The *VEGF-A* rs25648 ‘CC’ genotype was prognostic for improved PFS (HR 0.65, 95% CI 0.49 to 0.85; *P* = 0.002) and OS (HR 0.70, 95% CI 0.52 to 0.94; *P* = 0.019). The *VEGF-A* rs699947 ‘AA’ genotype was prognostic for shorter PFS (HR 1.32, 95% CI 1.002 to 1.74; *P* = 0.048). None of the analysed SNPs were predictive of bevacizumab efficacy outcomes. *VEGFR2* rs11133360 ‘TT’ was associated with a lower risk of grade ≥ 3 hypertension (*P* = 0.028). SNPs in *VEGF-A, VEGFR1* and *VEGFR2* did not predict bevacizumab benefit. However, *VEGF-A* rs25648 and rs699947 were identified as novel prognostic biomarkers and *VEGFR2* rs11133360 was associated with less grade ≥ 3 hypertension.

## Introduction

Bevacizumab, a recombinant humanised anti-vascular endothelial growth factor-A (VEGF-A) monoclonal antibody, is an approved anti-angiogenic drug which is used in combination with chemotherapy for the treatment of metastatic colorectal cancer (mCRC). Although it is an active and effective drug in this setting, the survival benefit in unselected populations is modest^1–3^ and it is associated with potential side effects. There is a need to define predictive biomarkers that can identify the patients who are most likely to derive benefit.

Bevacizumab exerts its effects by targeting host-mediated angiogenesis^4^. Its target, VEGF-A, is the most well-characterised pro-angiogenic ligand in the VEGF family^5^. Effects of VEGF-A on endothelial cell differentiation, proliferation, migration and vasculogenesis are primarily mediated by the receptor tyrosine kinase VEGFR2 (*KDR*)^6^, however VEGF-A also binds to VEGFR1 (*FLT-1*) with high affinity^7^ and induces weak kinase activity of this receptor^8^. Therefore, common germline genetic variability in *VEGF-A*, *VEGFR1*, and *VEGFR2* due to functional single nucleotide polymorphisms (SNPs) may explain heterogeneity in efficacy and toxicity amongst patients treated with bevacizumab.

Previous biomarker studies have identified a range of different single nucleotide polymorphisms (SNPs) in *VEGF-A, VEGFR1* and *VEGFR2* that were associated with efficacy outcomes (including objective response rate (ORR), progression-free survival (PFS) and overall survival (OS)) in studies of bevacizumab in mCRC. These include rs3025039^9^, rs2010963^9–11^, rs1570360^11^, rs699947^10^, rs833061^10,12^, rs25648^13^ (*VEGF-A*); rs9582036^14^, rs9513070^12^ (*VEGFR1*); rs2305948^15^, and rs12505758^16^ (*VEGFR2*). Other SNPs in *VEGF* family genes have also been associated with efficacy outcomes in studies of bevacizumab in non-CRC tumours, including rs699946^17,18^ (*VEGF-A*); rs9554316^19,20^, rs9513070^19^, rs9554320^19^, rs7993418^19^ (*VEGFR1*); rs2071559^21^ and rs11133360^18,22^ (*VEGFR2*). However, whilst some of these associations were independently verified in one or more additional studies, other studies showed no association or conflicting results. Notably, only one of the mCRC studies (ITACa)^9^ was a Phase III randomised controlled trial with a non-bevacizumab control arm, enabling distinction between prognostic and predictive effects. Recently, SNPs in multiple non-*VEGF* family genes have been found to be associated with efficacy outcomes in studies of bevacizumab in mCRC, with the results replicated in validation cohorts. The AA genotype for the rs8602 SNP in *MKNK1*, which is associated with upregulation of angiogenic factors and stimulation of angiogenesis, was associated with a shorter PFS compared to those with any C allele in patients with *KRAS* exon 2 wild type (wt) mCRC treated with FOLFIRI/Bevacizumab^23^. The AA genotype for the rs4588 SNP in *GC*, a gene encoding a vitamin D-binding protein, was associated with worse OS compared to those with any C allele in patients with mCRC treated with the same combination^24^. The G allele for the rs555008 SNP in *RSPO2*, one of the genes encoding R-spondin proteins which stimulate Wnt signalling, was associated with longer OS compared to the TT genotype in patients with *RAS* and *KRAS* wt mCRC^25^. *P* values for SNP by treatment interaction were not reported in these studies.

Hypertension is a common side effect which is observed in approximately a quarter of bevacizumab-treated patients^26^. Potential mechanisms include vasoconstriction due to decreased nitrous oxide (NO) production^27^, increased vascular tone secondary to inhibition of VEGF-mediated vasodilation^28^, microvascular rarefaction^29^, and renal dysfunction as a direct consequence of VEGF inhibition^30^ or indirectly through associated NO downregulation^31^.

Previous studies conducted in a range of tumour types have found associations between SNPs in *VEGF-A* (rs2010963^32,33^, rs833061^32,34^, rs699947^34^, rs3025039^34,35^) and *VEGFR2* (rs2305949^36^, rs1870377^37^) and bevacizumab-related hypertension. However, no association was replicated consistently across multiple studies and findings in several studies were discordant. SNPs in non-*VEGF* family genes were also recently reported to be associated with hypertension in studies of bevacizumab in mCRC. rs9381299 and rs834576 in the genomic region between *SLC29A1* and *HSP90AB1* were associated with early grade ≥ 3 hypertension, potentially through effects on adenosine signalling^38^. rs1129660 in *FIP200*, an autophagy-related gene, was associated with grade 2–3 hypertension in patients treated with FOLFIRI/bevacizumab^39^.

The Australian Gastro-intestinal Trials Group (AGITG) MAX clinical trial (NCT00294359) was an investigator-initiated phase III randomised controlled trial of first-line treatment for mCRC which met its primary end-point of improved PFS with the addition of bevacizumab to capecitabine-based chemotherapy^3^. The inclusion of a non-bevacizumab control arm provides an ideal dataset for retrospective predictive biomarker analysis. In this exploratory study, we examined the association of SNPs in *VEGF-A*, *VEGFR1*, and *VEGFR2* with prognosis, benefit from bevacizumab treatment and hypertension in patients receiving capecitabine alone and in combination with bevacizumab (± mitomycin C) in the MAX clinical trial.

## Material and methods

### Patients and study design

The study design and eligibility criteria for the MAX clinical trial have been reported previously^3^. The primary objective of the trial was to determine the effect on PFS of adding bevacizumab, with or without mitomycin, to first-line capecitabine monotherapy in patients with unresectable mCRC. Overall survival (OS) was a secondary end-point. Patients were enrolled from July 2005 to June 2007 and randomly assigned 1:1:1 to capecitabine (C), capecitabine plus bevacizumab (CB), or capecitabine, bevacizumab and mitomycin (CBM). Participants were evaluated for tumour response every 6 weeks on study. In the absence of significant toxicity, treatment was continued until confirmed disease progression.

### Ethics approval and consent to participate

Ethics approval for this study was obtained centrally from the Austin Health Human Research Ethics Committee. All participants in the current study provided written informed consent to participate in the main MAX clinical trial and additional informed consent for their tissue samples to be used in biomarker studies. The study was performed in accordance with the Declaration of Helsinki.

### Archival tissue collection and processing

Archived formalin-fixed paraffin-embedded (FFPE) tissue specimens collected at the time of diagnosis and associated histopathology reports were retrieved from the pathology departments where the diagnosis was made, and stored centrally. Personnel who performed histopathology reporting and scientific assays for biomarker analysis were blinded to patient identification and clinical outcomes.

Where available, a core of normal, colorectal adenoma, or colorectal tumour tissue (listed in order of preference) was extracted using a Beecher Mark II Tissue Arrayer (Beecher Instruments, Sun Prairie, WI, USA). Haematoxylin and eosin stained slides were used to guide the coring procedure.

For the first fifty cases (ordered consecutively by patient ID) where both colorectal tumour tissue and adjacent normal colorectal epithelial tissue were available, matching cores of tumour and normal tissue were extracted.

### SNP genotyping of tissue samples

Genomic DNA was extracted from the tissue cores using the QIAamp DNA FFPE Tissue Kit (Qiagen, Hilden, Germany), as per the manufacturer’s instructions. DNA concentration was determined using the NanoDrop 2000 Spectrophotometer (Thermo Fisher Scientific, Waltham, MA, USA).

All of the tested candidate SNPs in *VEGF-A*, *VEGFR1*, and *VEGFR2* were previously reported to have associations with clinical outcomes and/or to have an effect on corresponding VEGF ligand or receptor expression or function^10,17,40–47^. SNP genotyping was performed using Taqman SNP Genotyping Assays (Applied Biosystems, Waltham, MA, USA). Context sequences and primer sequences for the Taqman SNP Genotyping Assays used are provided in Supplementary Table S1. SNPs were required to have a minor allele frequency (MAF) of 10% or more to provide reassurance that they would have sufficient impact as a potential biomarker at the population level.

For each SNP assay, 5 ng of genomic DNA from each trial participant was diluted in 2.25 μL nuclease-free water, and mixed with 2.75 μL of reaction mix consisting of 2X Taqman GTXpress Master Mix (Applied Biosystems) and 20X TaqMan Genotyping Assay mix (Applied Biosystems). Samples were loaded into MicroAmp Optical 384-Well Reaction Plates with Barcode (Applied Biosystems), with each plate including at least one non-template control of nuclease-free water. Reaction plates were vortexed, centrifuged and PCR reactions performed on the ViiA 7 Real-Time PCR System (Applied Biosystems) with the following conditions: (1) a pre-read stage at 60 °C for 30 s, (2) a hold stage with an initial DNA polymerase activation step (95 °C for 20 s), (3) a PCR stage with 40 to 60 amplification cycles using a two-step cycling protocol of denaturing at 95 °C for 1 s and annealing and extension at 60 °C for 20 s, and (4) a post-read stage at 60 °C for 30 s.

Experiments were analysed using the Applied Biosystems ViiA 7 Real-Time PCR System software (Applied Biosystems). The genotyping optimisation tool was used to reveal traces to determine the ideal cycle for genotyping. Genotyping experiments were performed in duplicate as a minimum.

Each SNP was tested for departure from Hardy Weinberg Equilibrium (HWE) using the exact test in Haploview 4.2 (Broad Institute, Cambridge, MA, USA), the null hypothesis being that the genotype is in HWE in the population. A Bonferroni correction was applied to a significance level of *P* > 0.05, giving a significance level of *P* > 0.0024. SNPs with significant departure from HWE (*P* < 0.0024) were excluded from further analyses. The MAF for each SNP was calculated using Haploview 4.2.

### VEGF family gene and protein expression analysis

To examine whether specific *VEGF-A* SNP genotypes were associated with *VEGF-A* expression, we interrogated corresponding gene expression and pro-angiogenic protein expression data we had previously generated for this patient cohort^48,49^.

In brief, for gene expression analysis H&E stained sections were reviewed by a pathologist and macro-dissected to obtain tumour-rich tissue. RNA was extracted and converted into cDNA and gene expression profiling performed using the Almac Xcel Array (Almac, Craigavon, UK), utilising Affymetrix GeneChip technology optimised for use with FFPE tissue.

For analysis of protein expression levels, protein lysates were prepared from the FFPE tumour sections and quantified using the EZQ Protein Quantitation Kit (Life Technologies, Carlsbad, CA, USA). Protein lysates were assayed for expression of multiple proteins including VEGF-A, using a custom multiplex Bio-Plex array on the Bio-Plex 200 System instrument (Bio-Rad, Hercules, CA, USA).

### Statistical methods

Statistical analyses were performed using the statistical package ACCoRD (Analysis of Censored and Correlated Data) version 8.58 (Boffin Software, NSW, Australia) and Stata 13.1 (StataCorp, College Station, TX, USA). All reported *P* values are two-sided.

For the fifty consecutive cases where SNP genotyping was performed on matched tumour tissue and adjacent normal tissue, statistical analysis of concordance in the frequencies of each genotype between the tumour and normal tissue groups was calculated using Kendall’s τ. Concordance was analysed in cases where the full genotypes for both tumour and normal tissue had been determined.

Statistical analysis for PFS and OS was performed using the proportional hazards model. PFS was defined as time from randomisation until documented evidence of disease progression according to Response Evaluation Criteria in Solid Tumours (RECIST) version 1.0 or death as a result of any cause. OS was defined as the time from randomisation until death from any cause.

For each SNP, we first compared survival outcomes in participants with and without a homozygous major allele genotype. Survival outcomes in participants with and without a homozygous minor allele genotype were subsequently compared.

To determine whether SNP genotype was a predictive biomarker for resistance to bevacizumab, SNPs which showed a univariate association with survival outcomes were included in a model which included the allocated treatment covariate (‘CB and CBM’ versus ‘C’) and a ‘SNP by treatment’ interaction. These SNPs were also tested in a multivariate model which included baseline variables which were found to be associated with survival outcomes in the main MAX study (treatment received, Eastern Cooperative Oncology Group (ECOG) performance status, neutrophils, bilirubin and alkaline phosphatase levels, prior radiotherapy, whether the primary tumour was resected and the number of metastatic sites)^3^. Variables significantly associated with the survival outcomes in the multivariate model were retained in the final model.

In additional analyses, a multivariate model which included *KRAS* and *BRAF* mutation status along with the baseline prognostic variables was tested. Mutation status was only retained as a variable in the final model if it was significantly associated with survival outcomes.

A logistic regression model with SNP genotype status, treatment group covariate, and a ‘SNP-by-treatment’ interaction term was used to determine whether SNP genotype was predictive of an effect of bevacizumab on objective response rate (ORR) (defined as a best response of complete response (CR) or partial response (PR)).

For SNPs which were found to be associated with survival outcomes, the association between SNP genotype and corresponding (1) log2 transformed gene expression data from the Almac Xcel microarray and (2) protein expression data from Bioplex suspension array analyses were analysed using two-sided unpaired t-tests.

Univariate and multivariate associations between the SNPs and on-study grade ≥ 3 hypertension according to the National Cancer Institute Common Terminology Criteria for Adverse Events (NCI-CTCAE) version 3.0 were examined using logistic regression models. Models with SNP genotype alone, and with both the SNP genotype and allocated treatment covariates (‘CB and CBM’ versus ‘C’) were used to explore potential associations with hypertension, and both unadjusted and adjusted odds ratios (ORs) were reported. If the ORs were not estimable due to zero events in one cell of the 2 × 2 contingency table, the *P* values for the Conditional Binomial Exact Test (CBET) for independence between SNP and hypertension were reported^50^.

For each SNP, only participants with the full genotype identified were included in analyses of associations between SNP genotype and efficacy and hypertension outcomes.

Due to the exploratory nature of this analysis, no formal adjustment for multiple comparisons was made.

## Results

### Patient characteristics

**Figure 1:**
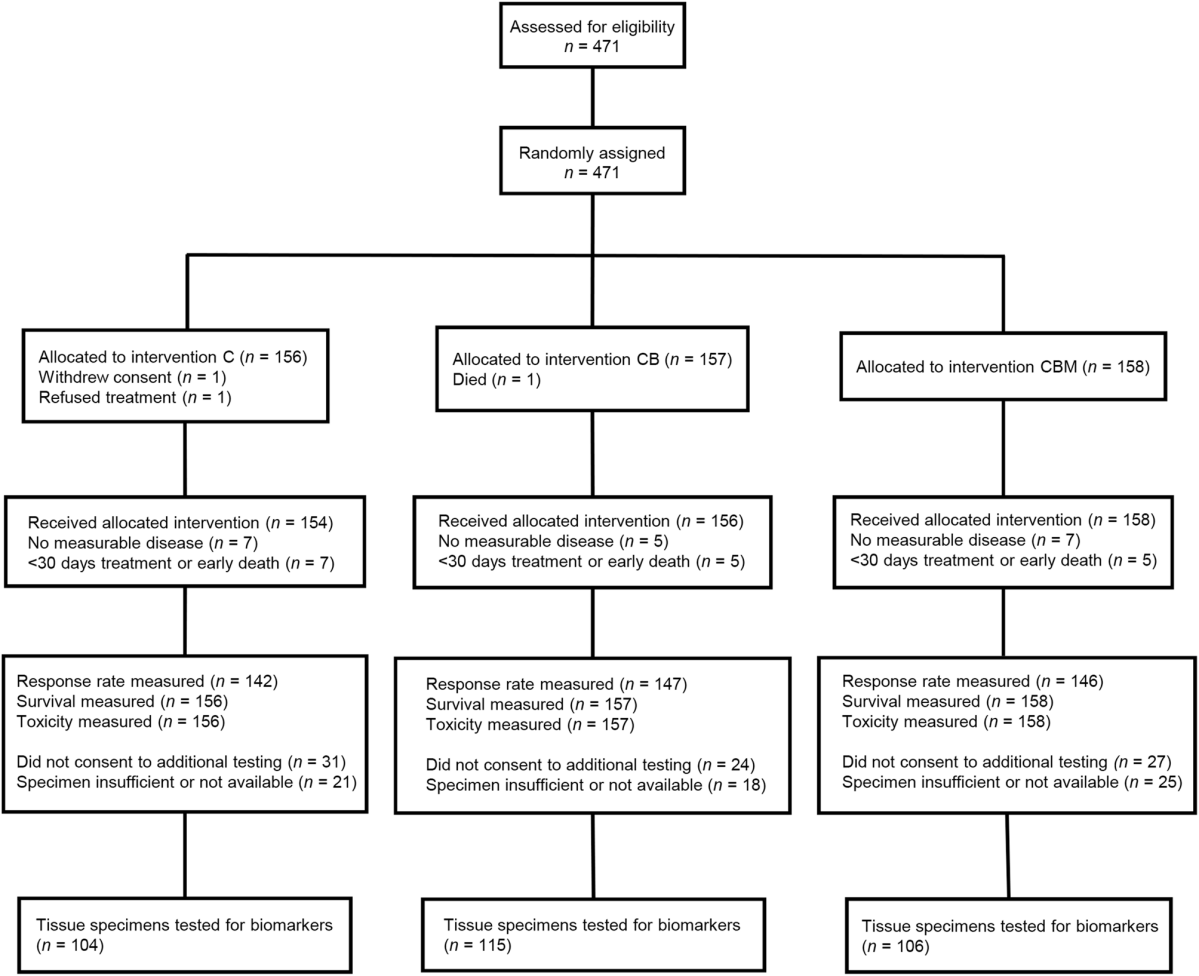
CONSORT flow diagram. Flow diagram of progress through the MAX clinical trial and VEGF SNP biomarker sub-study.

389 of the 471 randomised participants in the MAX clinical trial consented to participation in biological sub-studies. Of these, archived FFPE specimens were available from 325 participants (69% of the total study population) for analysis of *VEGF* family SNPs (Fig. 1).

For the remaining participants, tissue either could not be retrieved or was insufficient for extracting cores. The baseline characteristics and survival outcomes in the total study population and the SNP biomarker study population were comparable (Supplementary Table S2). The median follow-up time for the included participants was 30 months (range, 0.4 to 42.4 months).

### SNP genotyping

Of the available archived FFPE specimens from 325 participants, normal tissue was available for 164 participants (51%). Colorectal adenoma tissue was available for seven participants (2%). Colorectal carcinoma tissue was available for the remaining 154 participants (47%), of which 90% (*n* = 137) were from primary tumour (Supplementary Fig. S1).

Twenty-one SNPs were genotyped—7 in *VEGF-A* (rs699946, rs699947, rs833061, rs1570360, rs2010963, rs25648, rs3025039), 5 in *VEGFR1* (rs9513070, rs9554316, rs7993418, rs9582036, rs9554320) and 9 in *VEGFR2* (rs12505758, rs7655964, rs1870377, rs2305948, rs2305949, rs11133360, rs7667298, rs2071559, rs1551641) (Supplementary Table S1). Of these, 16 SNPs were found to be in HWE using Haploview and were used in subsequent analyses (Supplementary Table S3). The calculated MAF for each of these analysed SNPs was 10% or greater (Supplementary Table S4).

The full genotype was successfully identified in ≥ 84% of cases for each of the SNPs genotyped. The proportion of cases where the partial genotype was known (presence of the major allele or minor allele known, but only one allele determined) was not more than 7% for each SNP. The proportion of cases with an undetermined genotype (due to discordant genotyping calls between replicates, failure to amplify, or insufficient genomic DNA template) was not more than 9% for each SNP (Supplementary Table S5).

In the fifty cases of matched normal tissue and adjacent tumour tissue which we examined, concordance of genotype calls was strong (Kendall’s τ > 0.70; 15/16 SNPs) or moderate (Kendall’s τ > 0.40; 1/16 SNPs—rs12505758) for all SNPs analysed (Supplementary Table S6).

### Progression-free survival

**Figure 2:**
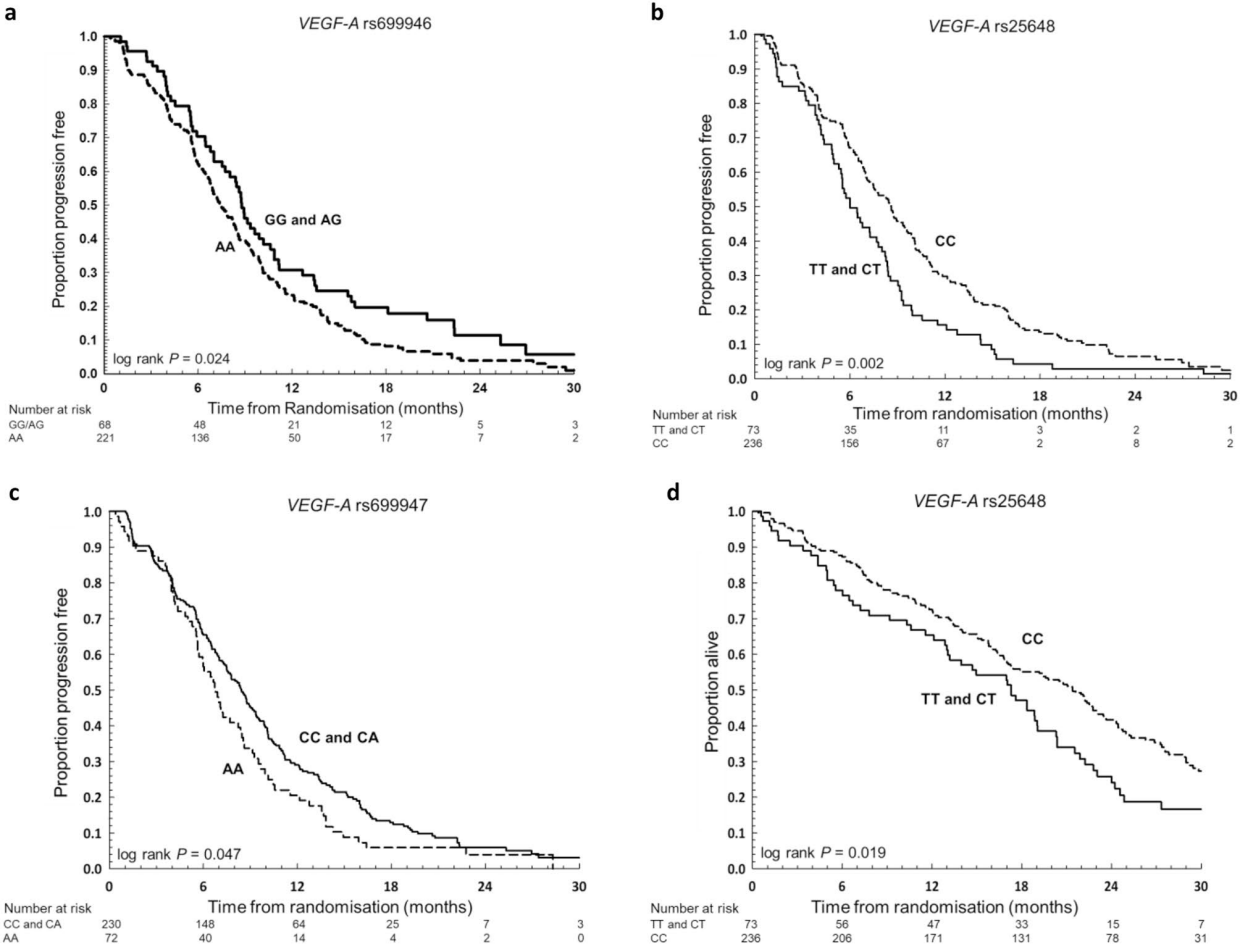
Survival outcomes by SNP genotypes. Progression free survival for patients with (**a**) *VEGF-A* rs 699946 ‘AA’ compared to ‘GG and AG’ genotypes, (**b**) *VEGF-A* rs25648 ‘CC’ compared to ‘TT and CT’ genotypes and (**c**) *VEGF-A* rs699947 ‘AA’ compared to ‘CC and AC’ genotypes. (**d**) Overall survival for patients with *VEGF-A* rs25648 ‘CC’ compared to ‘TT and CT’ genotypes. The *P*-value shown is for the log rank test of equality across the strata for the genotypes being compared.

**Figure 3:**
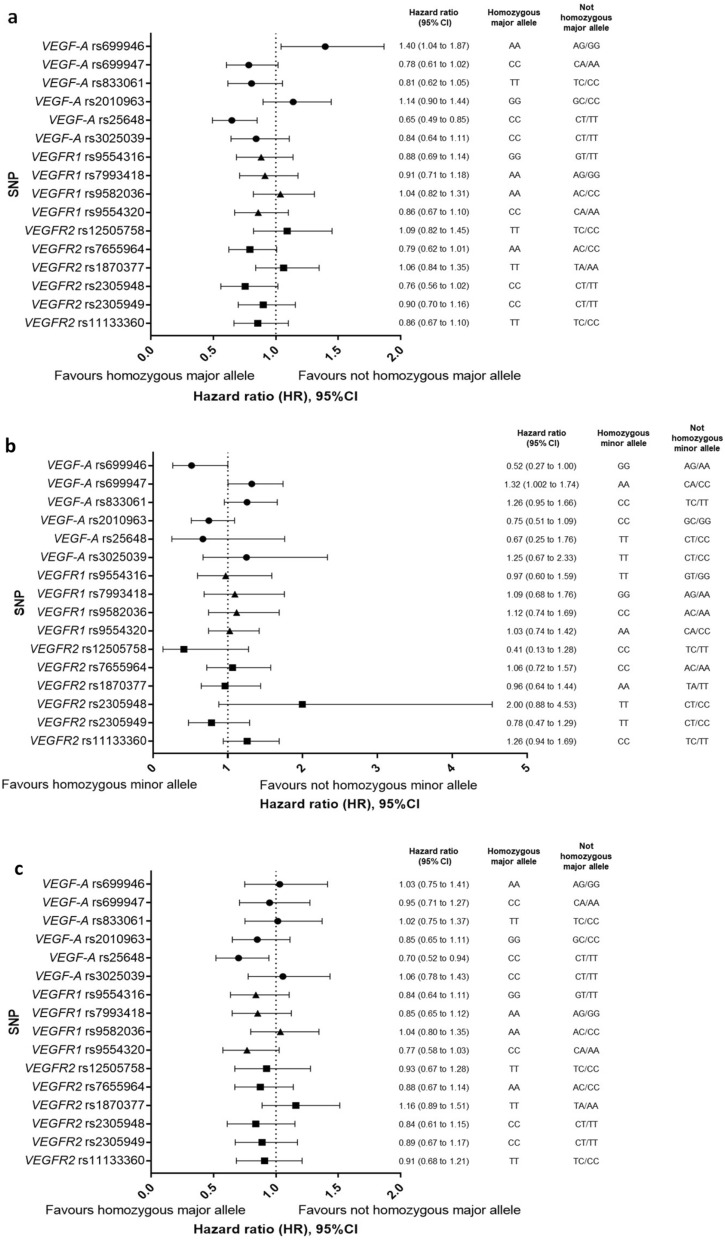
Forest plots of survival outcomes by SNP genotype. Hazard Ratios with 95% confidence intervals for the comparisons of (**a**) Progression free survival in patients with homozygous major allele genotype versus without a homozygous major allele genotype, (**b**) Progression free survival in patients with homozygous minor allele genotype versus without a homozygous minor allele genotype, and (**c**) Overall survival in patients with a homozygous major allele genotype versus without a homozygous major allele genotype.

There was a significantly lower PFS in participants with the *VEGF-A* rs699946 ‘AA’ genotype (homozygous major allele) compared to ‘GG and AG’ genotypes (median 7.5 months *versus* 8.8 months, log rank *P* = 0.024) (Fig. 2A). The hazard ratio (HR) for PFS in *VEGF-A* rs699946 ‘AA’ *versus* ‘GG and AG’ was 1.40 (95% CI 1.04 to 1.87, *P* = 0.025) (Fig. 3A, Table 1, Supplementary Table S7). A model including *VEGF-A* rs699946 genotype and allocated treatment (‘CB/CBM’ versus ‘C’) together with an interaction term (SNPxTreatment) did not show any statistically significant interaction effect between *VEGF-A* rs699946 genotype and bevacizumab treatment (interaction *P* value = 0.93).

**Table 1:** Prognostic associations of *VEGF-A* family SNP genotypes for progression free survival and overall survival.

Progression free survival (PFS)							
SNP		Univariate analysis		Variables in multivariable model		Multivariate analysis	
		HR (95% CI)	*P* value			HR (95% CI)	*P* value
***VEGF-A* rs699946**	Homozygous major allele ‘AA’ *vs* ‘GG and AG’	**1.40 (1.04 to 1.87)**	***P* = 0.025**				
***VEGF-A* rs25648**	**Homozygous major allele ‘CC’ *vs* ‘TT and CT’**	0.65 (0.49 to 0.85)	*P* = 0.002	***VEGF-A* rs25648**	**Homozygous major allele ‘CC’ *vs* ‘TT and CT’**	0.62 (0.47 to 0.82)	*P* = 0.001
				Treatment	**‘CB + CBM’ *vs* ‘C’**	0.59 (0.46 to 0.77)	*P* < 0.001
				ECOG PS	** ≥ 1 *vs* 0**	1.63 (1.34 to 1.99)	*P* < 0.001
				Neutrophils ≥ 8 × 10^9^/L	Yes *vs* no	1.60 (1.14 to 2.26)	*P* = 0.007
				Serum bilirubin ≥ 14 μmol/L	**Yes *vs* no**	1.53 (1.13 to 2.06)	*P* = 0.006
***VEGF-A* rs699947**	Homozygous minor allele ‘AA’ *vs* ‘CC and CA’	1.32 (1.002 to 1.74)	*P* = 0.048	***VEGF-A* rs699947**	Homozygous minor allele ‘AA’ *vs* ‘CC and CA’	1.50 (1.13 to 1.99)	*P* = 0.005
				Treatment	‘CB + CBM’ *vs* ‘C’	0.62 (0.48 to 0.80)	*P* < 0.001
				ECOG PS	≥ 1 *vs* 0	1.69 (1.39 to 2.07)	*P* < 0.001
				Neutrophils ≥ 8 × 10^9^/L	Yes *vs* no	1.60 (1.14 to 2.25)	*P* = 0.007
				Serum bilirubin ≥ 14 μmol/L	**Yes *vs* no**	1.53 (1.12—2.08)	*P* = 0.007

Overall survival (OS)							
SNP		Univariate analysis		Variables in multivariable model		Multivariate analysis	
		HR (95% CI)	*P* value			HR (95% CI)	*P* value
*VEGF-A* rs25648	Homozygous major allele ‘CC’ *vs* ‘TT and CT’	**0.70 (0.52 to 0.94)**	***P* = 0.019**	*VEGF-A* rs25648			
				*VEGF-A* rs25648	Homozygous major allele ‘CC’ *vs* ‘TT and CT’	0.68 (0.50 to 0.92)	*P* = 0.011
				ECOG PS	≥ 1 *vs* 0	2.08 (1.68 to 2.58)	*P* < 0.001
				Neutrophils ≥ 8 × 10^9^/L	Yes *vs* no	1.85 (1.27 to 2.70)	*P* = 0.001
				Serum ALP ≥ 140 U/L	Yes *vs* no	1.002 (1.000 to 1.003)	*P* = 0.028
				***VEGF-A* rs25648, ALP excluded from model**			
				*VEGF-A* rs25648	Homozygous major allele ‘CC’ *vs* ‘TT and CT’	0.67 (0.49 to 0.90)	*P* = 0.008
				ECOG PS	≥ 1 *vs* 0	2.07 (1.66 to 2.56)	*P* < 0.001
				Neutrophils ≥ 8 × 10^9^/L	Yes *vs* no	1.84 (1.27 to 2.68)	*P* = 0.001
				***VEGF-A* rs25648, *BRAF* included in model**			
				*VEGF-A* rs25648	Homozygous major allele ‘CC’ *vs* ‘TT and CT’	0.70 (0.52 to 0.96)	*P* = 0.024
				ECOG PS	≥ 1 *vs* 0	2.10 (1.68 to 2.61)	*P* < 0.001
				Neutrophils ≥ 8 × 10^9^/L	Yes *vs* no	1.85 (1.26 to 2.72)	*P* = 0.002
				*BRAF* mutation status	Wild type *vs* mutant	0.49 (0.32 to 0.75)	*P* = 0.001

*VEGF-A* = *vascular endothelial growth factor-A*; SNP = single nucleotide polymorphism; HR = hazard ratio; CI = confidence interval; CB = capecitabine and bevacizumab; CBM = capecitabine, bevacizumab and mitomycin; C = capecitabine; ECOG PS = Eastern Cooperative Oncology Group performance status; ALP: alkaline phosphatase.

Participants with the *VEGF-A* rs25648 ‘CC’ genotype (homozygous major allele) had a significantly longer PFS compared to the ‘TT and CT’ genotypes (median 8.6 months *versus* 6.0 months, log rank *P* = 0.002) (Fig. 2B). The HR for PFS in *VEGF-A* rs25648 ‘CC’ *versus* ‘TT and CT’ was 0.65 (95% CI 0.49 to 0.85, *P* = 0.002) (Fig. 3A, Table 1, Supplementary Table S7). A similar model to the analysis of *VEGF-A* rs699946 was fitted to the *VEGF-A* rs25648 genotype, and no statistically significant interaction was observed between this SNP and the addition of bevacizumab (interaction *P* value = 0.78).

Participants with the *VEGF-A* rs699947 ‘AA’ genotype (homozygous minor allele) had a significantly inferior PFS compared to the ‘CC and CA’ genotypes (median 6.8 months *versus* 8.4 months, log rank *P* = 0.047) (Fig. 2C). The HR for PFS in *VEGF-A* rs699947 ‘AA’ *versus* ‘CC and CA’ was 1.32 (95% CI 1.002 to 1.74, *P* = 0.048) (Fig. 3B, Table 1, Supplementary Table S7). As for *VEGF-A* rs699946 and *VEGF-A* rs25648, a model was fitted to examine interaction effects for *VEGF-A* rs699947 genotype and the addition of bevacizumab. No statistically significant interaction effect was observed (interaction *P* value = 0.70).

In a multivariable model adjusting for baseline prognostic factors, of the SNPs analysed, only *VEGF-A* rs25648 and *VEGF-A* rs699947 remained significant independent prognostic factors for PFS. The adjusted HR for *VEGF-A* rs25648 ‘CC’ *versus* ‘TT and CT’ was 0.62 (95% CI 0.47 to 0.82, *P* = 0.001) and the adjusted HR for *VEGF-A* rs699947 ‘AA’ *versus* ‘CC and CA’ was 1.50 (95% CI 1.13 to 1.99, *P* = 0.005) (Table 1). In an additional multivariable model which also included *KRAS* and *BRAF* mutation status, neither of these covariates was significantly associated with the PFS outcome.

### Overall survival

There was a significantly longer OS in participants with the *VEGF-A* rs25648 ‘CC’ genotype (homozygous major allele) compared to the ‘TT and CT’ genotypes (median 21.4 months *versus* 17.3 months, log rank *P* = 0.019) (Fig. 2D). The HR for OS in *VEGF-A* rs25648 ‘CC’ *versus* ‘TT and CT’ was 0.70 (95% CI 0.52 to 0.94, *P* = 0.019) (Fig. 3C, Table 1, Supplementary Table S8). A model including *VEGF-A* rs25648 genotype and allocated treatment (‘CB/CBM’ versus ‘C’) together with an interaction term (SNPxTreatment) did not show any statistically significant interaction effect between *VEGF-A* rs25648 genotype and bevacizumab treatment (interaction *P* value = 0.54).

The *VEGF-A* rs25648 ‘CC’ genotype remained a significant prognostic factor for OS in a multivariable model adjusting for baseline prognostic factors. The adjusted HR for *VEGF-A* rs25648 ‘CC’ *versus* ‘TT and CT’ was 0.68 (95% CI 0.50 to 0.92, *P* = 0.011). In the multivariable model, the HR for serum alkaline phosphatase (ALP) was 1.002 (95% CI 1.000 to 1.003), which may not be considered clinically significant. When serum ALP was excluded from the multivariable model, the adjusted HR for OS was 0.67 (95% CI 0.49 to 0.90, *P* = 0.008) (Table 1).

In a further multivariable model which included *KRAS* and *BRAF* mutation status, *BRAF* but not *KRAS* mutation status was significantly associated with OS. When *BRAF* mutation status was additionally included in the final multivariable model, the adjusted HR for *VEGF-A* rs25648 ‘CC’ *versus* ‘TT and CT’ did not change appreciably (HR 0.70, 95% CI 0.52 to 0.96, *P* = 0.024) (Table 1).

### Objective response

Participants with *VEGF-A* rs699946 ‘GG and AG’, rs25648 ‘CC’ or rs699947 ‘CC and CA’ genotypes did not have a greater likelihood of achieving an objective response with the addition of bevacizumab, compared to the alternative genotypes (*P* = 0.63, *P* = 0.77, and *P* = 0.70 for the interaction between SNP genotype and the assigned treatment, respectively) (Table 2). None of the other analysed SNPs was predictive of improved objective response with the addition of bevacizumab (Supplementary Table S9).

**Table 2:** Objective response rate by *VEGF-A* rs699946, rs25648 and rs699947 genotypes.

*VEGF-A* 699946			
Treatment	AA genotype *n* (%)	GG and AG genotypes *n* (%)	*P* value^a^
C	26 (79)	7 (21)	0.63
CB	29 (78)	8 (22)	
CBM	31 (74)	11 (26)	

*VEGF-A* 25648			
Treatment	CC genotype *n* (%)	TT and CT genotypes *n* (%)	*P* value^a^
C	29 (83)	6 (17)	0.77
CB	29 (73)	11 (27)	
CBM	40 (87)	6 (13)	

*VEGF-A* 699947			
Treatment	AA genotype *n* (%)	CC and CA genotypes *n* (%)	*P* value^a^
C	9 (26)	26 (74)	0.70
CB	9 (24)	29 (76)	
CBM	8 (18)	37 (82)	

*VEGF-A* = *vascular endothelial growth factor-A*; C = capecitabine; CB = capecitabine and bevacizumab; CBM = capecitabine, bevacizumab and mitomycin.

^a^*P* value for interaction between SNP genotype biomarker status and the allocated treatment (‘CB + CBM’ *vs* ‘C’).

### SNP genotype and VEGF-A expression

Data from gene expression profiling was available for 239 of 309 (77%) participants who had a full genotype known for *VEGF-A* rs25648, and 234 of 302 (77%) participants who had a full genotype known for *VEGF-A* rs699947.

**Figure 4:**
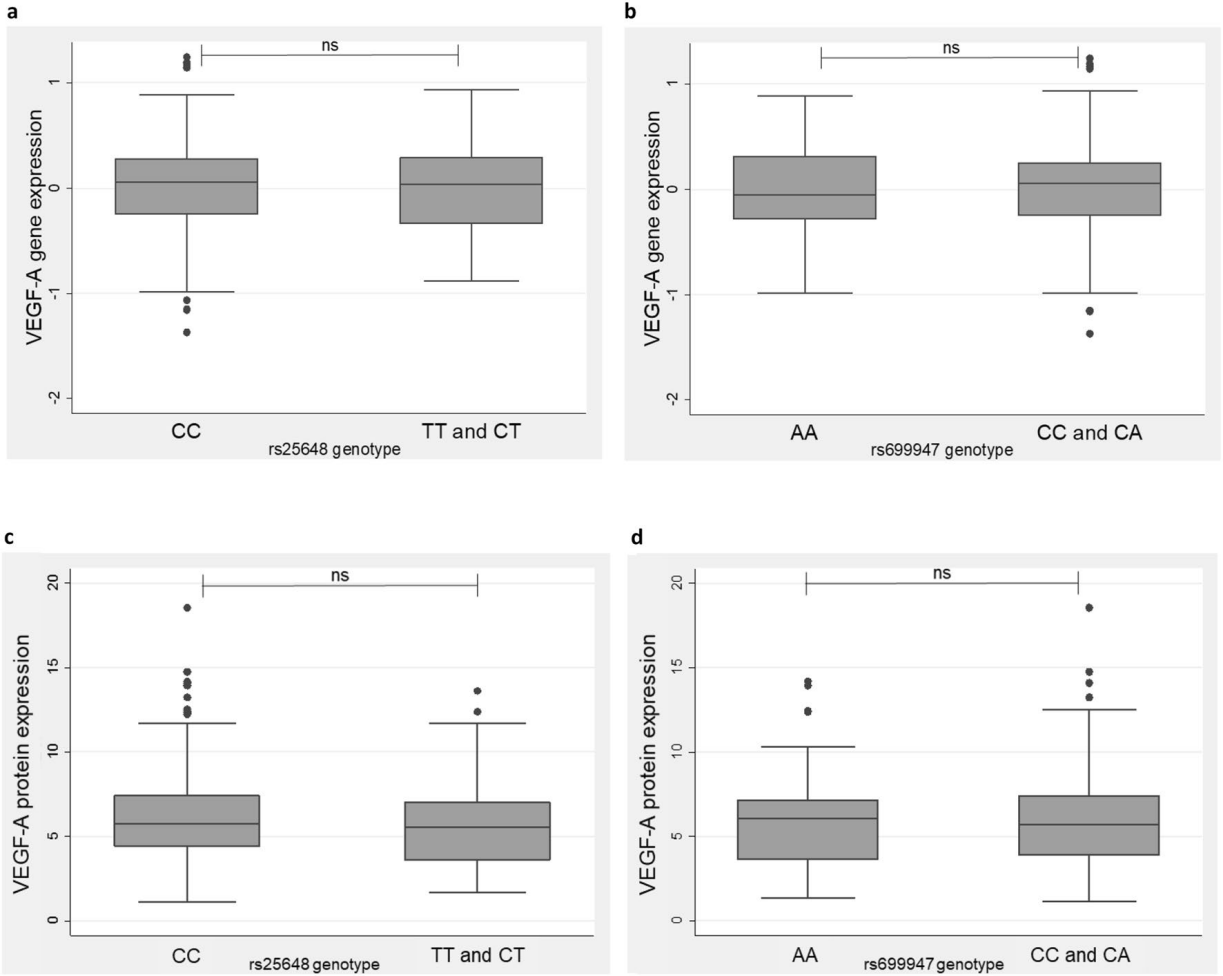
Box and whiskers plots of prognostic *VEGF-A* SNP genotypes and corresponding VEGF-A gene or protein expression. (**a**) *VEGF-A* rs25648 and (**b**) *VEGF-A* rs699947 genotypes with corresponding *VEGF-A* gene expression (log transformed, Almac Xcel microarray data). (**c**) *VEGF-A* rs25648 and (**d**) *VEGF-A* rs699947 genotypes with corresponding VEGF-A protein expression (Bioplex suspension array data). Boxes show the median with 25th (lower hinge) and 75th (upper hinge) percentiles, and whiskers show the upper and lower adjacent values. Solid circles represent the outliers.

However, no difference in *VEGF-A* gene expression level was observed between the better prognosis *VEGF-A* rs25648 ‘CC’ genotype group and the ‘TT and CT’ genotypes (mean 0.00091 *versus* − 0.020, *P* = 0.77). Similarly, there was no difference in *VEGF-A* gene expression level between the better prognosis *VEGF-A* rs699947 ‘CC and CA’ genotypes and the ‘AA’ genotype (mean 0.0012 *vs.* − 0.022, *P* = 0.74) (Fig. 4a,b).

Data from protein expression profiling was available for 193 of 309 (62%) participants who had a full genotype known for *VEGF-A* rs25648, and 193 of 302 (64%) participants who had a full genotype known for *VEGF-A* rs699947.

Consistent with the mRNA expression data, no difference in VEGF-A protein expression level was observed between the better prognosis *VEGF-A* rs25648 ‘CC’ genotype group and the ‘TT and CT’ genotypes (mean 6.26 *versus* 5.80, *P* = 0.38), and between the better prognosis *VEGF-A* rs699947 ‘CC and CA’ genotypes compared to the ‘AA’ genotype (mean 6.04 *versus* 6.26, *P* = 0.66) (Fig. 4c,d).

### Grade ≥ 3 hypertension

Participants with the *VEGFR2* rs11133360 ‘TT’ genotype (homozygous major allele) had a significantly lower odds of grade ≥ 3 hypertension than participants with the ‘CC and TC’ genotypes (*P* = 0.028, Table 3).

**Table 3:** Association between *VEGFR2* rs11133360 genotypes and grade ≥ 3 hypertension.

*VEGFR2* rs111333360	Grade ≥ 3 hypertension (*n*)		
	*No*	*Yes*	
***Not homozygous TT***	180	9	
***Homozygous TT***	102	0	*P* = 0.028

^a^Conditional Binomial Exact Test (CBET) for independence between SNP and hypertension.

As there were no events in participants with the *VEGFR2* rs11133360 ‘TT’ genotype, an OR was not estimable for both a univariate and multivariate analysis adjusting for allocated treatment. There was no association between any of the other SNPs and grade ≥ 3 hypertension (Supplementary Table S10).

## Discussion

In this study we interrogated genomic DNA from mCRC patients who participated in the phase III MAX clinical trial to identify polymorphisms in the *VEGF-A*, *VEGFR1* and *VEGFR2* genes which were prognostic or predictive of outcome following first-line chemotherapy with or without bevacizumab treatment.

We observed that the *VEGF-A* rs25648 ‘CC’ genotype was associated with improved PFS and OS outcomes, and the *VEGF-A* rs699947 ‘AA’ genotype was associated with shorter PFS. However, neither of these SNPs was predictive of greater bevacizumab treatment effect on PFS and OS, nor ORR. The *VEGFR2* rs11133360 ‘TT’ genotype was associated with a lower risk of grade ≥ 3 hypertension regardless of bevacizumab treatment.

The MAX study had the ideal study design for incorporation of a retrospective predictive biomarker study, as it was a Phase 3 randomised controlled trial which included a non-bevacizumab chemotherapy-only control arm. We identified only one other study which examined SNPs in *VEGF-A*, *VEGFR1* and *VEGFR2* as predictive biomarkers of bevacizumab outcome in patients with mCRC which also had this optimal study design (Ulivi et al.^9^). In contrast to our study, where SNP genotyping was performed using FFPE normal tissue and tumour tissue in 50% and 47% of participants respectively, Ulivi et al. used peripheral blood samples in 153/237 (65%) participants and FFPE tumour tissue in 84/237 (35%) participants. Their study also did not identify any SNPs which were predictive of bevacizumab response. However, they found that the *VEGF-A* rs3025039 ‘TT’ genotype was prognostic for poorer PFS and OS, and that the *VEGF-A* rs2010963 ‘GC’ genotype was prognostic for an inferior ORR. In comparison, there was no association between these SNP genotypes and efficacy outcomes in our study.

However, our study did have some limitations. As this was a *post-hoc* retrospective analysis of multiple genetic biomarkers, the findings are exploratory in nature. Therefore, there is a possibility that our findings may have been a chance finding. We did not make specific a priori hypotheses with respect to which SNPs would demonstrate clear signals, the magnitude of any signals observed, or the variability and the strength (*P* values) of these signals. Accordingly, due to the exploratory nature of the analysis, no formal adjustment for multiple comparisons was made. We have not confirmed our findings in a second validation cohort. While we considered such a test-validation approach, we were constrained by the distribution of events within SNP level, the distribution of treatment across the levels of SNPs, the lack of knowledge regarding which SNPs would hold the signals, and the relatively small sample size of the study.

This biomarker analysis is restricted to 69% of participants in the MAX study for whom FFPE tissue samples were available, and is therefore not necessarily generalisable to the general population. However, the baseline characteristics and survival outcomes in the total study population and the SNP biomarker study population were comparable. SNPs were genotyped using a candidate approach, based on previous demonstration of functional effects on corresponding gene and/or protein expression or associations with clinical outcomes. An alternative approach for selecting SNPs to genotype is SNP tagging. This is a more systematic method that may lead to the testing of SNPs which were not identified in previous studies^19^.

Genetic variants may be associated with different disease risk in different ethnic populations^51^, and race or ethnicity may be a confounder in SNP biomarker studies^20^. Heterogeneity of effect by race or ethnicity may be observed due to variation in linkage disequilibrium among populations, allelic heterogeneity, or gene–gene and gene-environment interactions^51–54^. Information on the race or ethnicity of participants was not collected in the MAX clinical trial, therefore, we were not able to restrict analyses to an ethnically homogenous population or adjust for ethnicity in the analysis, which are approaches which have been used in some previous genetic biomarker studies of bevacizumab^17,19,20,55,56^.

Our finding that the *VEGF-A* rs25648 ‘CC’ genotype was associated with improved PFS and OS is supported by the results of a phase II single-arm study of patients treated with first-line FOLFIRI and bevacizumab for mCRC (Becouarn et al.), where participants with *VEGF-A* rs25648 C-variants had a longer OS than those with alternative genotypes^13^.

Our finding that the *VEGF-A* rs699947 ‘AA’ genotype was prognostic for shorter PFS than C-variants in the MAX study is supported by one phase III study of patients treated with first-line docetaxel with or without bevacizumab for metastatic breast cancer, in which the *VEGF-A* rs699947 C-allele was associated with longer PFS in the placebo arm^55^. However, the results of several other previous studies in the literature conflict with this finding. In a single-arm pilot study of 59 patients with mCRC who were treated with second-line gemcitabine, irinotecan and bevacizumab, Abajo et al. reported that participants with at least one of the *VEGF-A* rs699947 ‘AA’, rs833061 ‘CC’, or rs2010963 ‘GG’ genotypes had a significantly longer median TTP than those without these genotypes^10^. In studies of patients with glioblastoma (Galanis et al.)^21^ and metastatic breast cancer (Schneider et al.)^32^, the *VEGF-A* rs699947 AA genotype was associated with a superior 6-month PFS and median OS, respectively. The conflicting results amongst these studies of *VEGF* family SNPs and efficacy outcomes could be explained by the heterogeneous patient populations, or differences in tumour vasculature between different cancer types or following treatment with different regimens^22^.

In this study, no participant with *VEGFR2* rs11133360 ‘TT’ recorded grade ≥ 3 hypertension. Due to no events in participants with the *VEGFR2* rs11133360 ‘TT’ genotype, an OR was not estimable for multivariate analysis adjusting for bevacizumab treatment. Further research validating this finding in a larger cohort of patients would support our findings and may enable further analysis adjusting for bevacizumab. To our knowledge, this is the first study to demonstrate an association between *VEGFR2* rs11133360 and hypertension, although a previous study showed an association between the rs11133360 T-allele and improved PFS in patients who received bevacizumab^18,22^. We did not find an association between other VEGF family SNPs and grade ≥ 3 hypertension.

Having identified associations between specific SNPs in *VEGF* family genes and efficacy outcomes, we considered how these SNPs may alter VEGF-A function to bring about this effect. One possibility is that, being located in the 5’UTR (rs25648) or near or within a proximal enhancer/promoter regulatory element (rs699947), these SNPs may impact levels of VEGF-A mRNA expression. However, no associations between these *VEGF-A* SNP genotypes and corresponding VEGF-A expression which could explain their links with prognosis were observed. Our group previously found that tumour expression of VEGF-D using immunohistochemistry may be a potential predictive biomarker for bevacizumab efficacy on PFS in patients with mCRC^57^. However, we did not analyse associations between SNPs in *VEGF-A* and its receptors and VEGF-D expression because they would not be expected to have an effect on VEGF-D expression or function. Notably, the FFPE tissue samples which we used to measure *VEGF-A* mRNA and protein expression were from participants’ primary tumours. The majority of patients did not have tissue samples from their metastatic tumour available for mRNA extraction—a common situation in biomarker studies in patients with metastatic disease^58^. As angiogenesis is a complex, dynamic and adaptive process which varies over time^22,58^, *VEGF-A* expression in the primary tumour may not reflect expression levels in the metastatic tumour at the time of study participation, when prognosis was examined. This may explain the observed lack of association between VEGF-A gene and protein expression and *VEGF-A* SNP genotypes. However, in 47 (10%) MAX study participants for whom matched primary and metastatic tumour samples were available, we have found a moderate positive correlation between *VEGF-A* mRNA expression in the primary and metastatic tumour (*r* = 0.40, *P* = 0.006). Studies examining the association between VEGF-A mRNA in matched primary and metastatic tumour from a larger number of patients are required to further address this.

Another possibility is that the SNPs which we identified as prognostic biomarkers exert their prognostic impact by affecting the expression of other genes, for example, by acting as a distal enhancer.

rs11133360, which was associated with hypertension in our study, is located in the third intron of the *VEGFR2* gene, ~ 7 kb from the promoter and 5’UTR (Supplementary Fig. S2). Intronic SNPs may influence the transcriptional activity or splicing efficiency of host genes, or alter the expression of alternative transcripts^59^. Notably, our bioinformatic analysis revealed that rs11133360 is located within a GATA2 binding region and also coincides with a DNAase I hypersensitivity site (Supplementary Fig. S2). GATA2 has previously been demonstrated to be a bona fide transcription factor that regulates the expression of mouse isoforms of *VEGFR2* (*FLK-1*) via an enhancer sequence^60^. Our in silico analysis indicated that the ‘not homozygous TT’ (i.e. ‘CC’ or ‘CT’) genotype is expected to result in the loss of GATA2 binding at the rs11133360-associated GATA2 binding site, which could lead to the reduction of GATA2-mediated *VEGFR2* expression in endothelial cells. Blocking of VEGFR2 has been associated with hypertension in pre-clinical studies^61^, providing biological plausibility for our finding of increased hypertension in patients without the homozygous ‘TT’ genotype.

## Conclusions

In conclusion, we did not find any grade ≥ 3 hypertension among participants having the *VEGFR2* rs11133360 ‘TT’ genoptype. This is the first study to demonstrate this association and future research should focus on validating this finding in further cohorts. We also found that the *VEGF-A* rs25648 ‘CC’ genotype was a prognostic biomarker for improved PFS and OS outcomes, and the *VEGF-A* rs699947 ‘AA’ genotype was prognostic for a shorter PFS. However, neither of these SNPs were predictive biomarkers for bevacizumab treatment effect. Ongoing research to identify and validate predictive biomarkers for efficacy outcomes in patients with mCRC treated with bevacizumab is required, in order to better select patients for this treatment.

## Supplementary Information

Supplementary Information.

## Data Availability

The datasets generated during and/or analysed during the current study are available from the corresponding author on reasonable request.
