# Supplementary material for: VEGF-A, VEGFR1 and VEGFR2 single nucleotide polymorphisms and outcomes from the AGITG MAX trial of capecitabine, bevacizumab and mitomycin C in metastatic colorectal cancer
Source: Sci Rep. 2022 Jan 24;12:1238. doi: 10.1038/s41598-021-03952-y (PMC8786898; doi:10.1038/s41598-021-03952-y)
Supplement: Supplementary file 1 — Supplementary Information. [file 41598_2021_3952_MOESM1_ESM.pdf]

Title

VEGF-A, *VEGFR1* and *VEGFR2* single nucleotide polymorphisms and outcomes from the AGITG MAX trial of capecitabine, bevacizumab and mitomycin C in metastatic colorectal cancer

Authors

Fiona Chionh, Val Gebski, Sheren J Al-Obaidi, Jennifer K Mooi, Maressa A Bruhn, Chee K Lee, Anderly C Chüeh, David S Williams, Andrew J Weickhardt, Kate Wilson, Andrew M Scott, John Simes, Jennifer E Hardingham, Timothy J Price, John M Mariadason†, Niall C Tebbutt†

†These authors contributed equally to this work

Supplementary Information

Supplementary Tables	Page
**Table S1.** Context sequences and primer sequences for Taqman SNP genotyping assays	2
**Table S2.** Characteristics in the total and SNP biomarker study population	4
**Table S3.** Hardy Weinberg Equilibrium *P*-values for the 21 genotyped SNPs in *VEGF-A, VEGFR1 and VEGFR2*	5
**Table S4.** Location and Minor Allele Frequency of 16 analysed SNPs	6
**Table S5.** Results of SNP genotyping for 16 SNPs in *VEGF-A, VEGFR1 and VEGFR2*	7
**Table S6.** Concordance in SNP genotype between fifty cases of matched colorectal normal and tumour tissue	8
**Table S7.** Univariate associations between SNPs in *VEGF* family members and progression-free survival outcomes	9
**Table S8.** Univariate associations between SNPs in *VEGF* family members and overall survival outcomes	10
**Table S9.** Objective response rate by SNP genotypes in *VEGF-A, VEGFR1 and VEGFR2*	11
**Table S10.** Grade ≥ 3 hypertension by SNP genotypes in *VEGF-A, VEGFR1 and VEGFR2*	14

Supplementary Figures	Page
**Figure S1.** Characteristics of tissue used for SNP genotyping	15
**Figure S2.** Genomic location of *VEGFR2* rs11133360 and predicted functional effects on *VEGFR*2 expression	16

Table S1. Context sequences and primer sequences for Taqman SNP genotyping assays

Gene	SNP	Context sequence[a]/primer sequences	Taqman Assay ID
VEGF-A	rs699946	TTGTTTAAATTCTCTGACAGAGACA**[A/G]**ACTGT CCCGGGGGGCGGGGAAGGCA	C___1647395_10
	rs699947	GCCAGCTGTAGGCCAGACCCTGGCA**[A/C]**GATCT GGGTGGATAATCAGACTGAC	C___8311602_10
	rs833061	GAGTGTGTGCGTGTGGGGTTGAGGG**[C/T]**GTTGG AGCGGGGAGAAGGCCAGGGG	C___1647381_10
	rs1570360	Forward primer (5' to 3'): CGGGCCAGGCTTCACT<br>Reverse primer (5' to 3'): CCGCTACCAGCCGACTTTTAA<br>Reporter 1 (first allele/G, VIC): CTCAGCCCCTCCACAC<br>Reporter 2 (second allele/A, 6-FAM): CCTCAGCCCTTCCACAC	Not applicable, custom made
	rs2010963	CGCGCGGGCGTGCGAGCAGCGAAAG**[C/G]**GACA GGGGCAAAGTGAGTGACCTGC	C___8311614_10
	rs25648	GCCGCGCCGGCCCCGGTCGGGCCTC**[C/T]**GAAAC CATGAACTTTCTGCTGTCTT	C___791476_10
	rs3025039	Forward primer (5' to 3'): ACTCCGGCGGAAGCATTC<br>Reverse primer (5' to 3'):<br>AGCAAGAAAAATAAAATGGCGAATCCA<br>Reporter 1 (first allele/C, VIC):<br>CAAGAGGGACCGTGCTG<br>Reporter 2 (second allele/T, 6-FAM):<br>AAGAGGGACCATGCTG	Not applicable, custom made
VEGFR1	rs9513070	TTGCCCAACTTGTGGCCCACGGGCT**[A/G]**CATGA ATCCCAGGATGGCTTTGAGT	C___30362252_10
	rs9554316	AAAGACATCATTCGATTTTTTTTCT**[G/T]**GAACTC AATGGAATTCTCAGCCCCC	C___32231224_20
	rs7993418	GGCTCATGAACTTGAAAGCATTTAC**[A/G]**TATCT AATGAAGAAACAGAAAGAAT	C___1910654_10
	rs9582036	TGTGCCCAGCAACAATAGCCTTCTT**[A/C]**CAAAA TGTATACTAAAGTATTTAGG	C___1910658_10
	rs9554320	TGTGTGTAGCTGATCATTCTCCCCC**[A/C]**GCACTG CAAAGCCGCTGTTACAAAC	C___32231227_10

Table S1. Context sequences and primer sequences for Taqman SNP genotyping assays *continued*

Gene	SNP	Context sequence/primer sequences[a]	Taqman Assay ID
VEGFR2	rs12505758	ATGTCAACATATCTCGATGCAATAC**[C/T]**TGGCA TCATAGATTTGGCAGAGTAA	C___1673865_10
	rs7655964	GTGTATATGTACCACATTTTTTTTT**[A/C]**TCCCAC TGACCTTCTATTATGAAAA	C__26111282_10
	rs1870377	GGTATGGGTTTGTCACTGAGACAGC**[A/T]**TGGCT ATAAGAAAGAGATAACAGCG	C__11895315_20
	rs2305948	TACAATCCTTGGTCACTCCGGGTTA**[C/T]**ACCATC TATAGTTAAGGTGCTCAAA	C__22271999_20
	rs2305949	CCCTAGAGCAAGTAAATTGAAAAAA**[C/T]**AGAA CATGAGAGAGCAAATAAGCCT	C__16192173_20
	rs11133360	TTCACATTGCTATGCCCAACACATC**[C/T]**CATCA AGCATTCTCAGAGTGCTTCA	C__26111278_10
	rs7667298	GCAGAGGAAACGCAGCGACCACACA**[C/T]**TGAC CGCTCTCCCGGGGTCCCGGGA	C__28992770_10
	rs2071559	GAAAACGCACTTGCCCAGTTCGCCA**[A/G]**CATTC CCGCTATTTCCCAAAATATT	C__15869271_10
	rs1551641	TTGGCAGAGTCTCAATGAAATCAGT**[C/T]**TACCA CATTCCCTCCCACACAGCAC	Not applicable, custom made

Abbreviations: SNP = single nucleotide polymorphism; *VEGF-A = vascular endothelial growth factor-A*; *VEGFR1 = vascular endothelial growth factor receptor 1*; *VEGFR2 = vascular endothelial growth factor receptor 2*

[a]Alleles **[1/2]** in the context sequences denote **[**VIC reporter-labelled Allele **1,** FAM reporter-labelled Allele **2]**

Table S2. Characteristics in the total and SNP biomarker study population

Characteristic	Total study population (*n* = 471)	SNP biomarker study population (*n* = 325)
**Age (years)**		
Median	67	68
Range	32 - 86	31 - 85
**Male (*n*, %)**	295 (63)	200 (62)
**ECOG performance status (*n*, %)**		
0	263 (56)	187 (58)
1	178 (38)	118 (36)
2	30 (6)	20 (6)
**Prior adjuvant treatment (*n*, %)**		
Chemotherapy	104 (22)	70 (22)
Radiotherapy	59 (13)	32 (10)
**Primary tumour location (*n*, %)**		
Right sided	124 (26)	94 (29)
Left sided	316 (67)	216 (66)
Unknown	31 (7)	15 (5)
**Primary tumour resected (*n*, %)**		
Yes	371 (79)	280 (86)
**Any metastases resected (*n*, %)**		
Yes	45 (10)	29 (9)
**Extent of disease at baseline (*n*, %)**		
Local disease (colon or rectum)	169 (36)	103 (32)
Liver metastases	353 (75)	240 (74)
Lymph node metastases	219 (46)	148 (46)
Lung metastases	185 (39)	126 (39)
Bone metastases	18 (4)	11 (3)
Peritoneal metastases	84 (18)	54 (17)
Other metastases	50 (11)	37 (11)
**Capecitabine dosage (*n*, %)**		
1 g/m^2 BD	314 (67)	208 (64)
1.25 g/m^2 BD	157 (33)	117 (36)
**Median PFS (months, 95% CI)**	7.2 (6.8 – 7.8)	7.8 (7.0 – 8.5)
**Median OS (months, 95% CI)**	17.9 (16.7 – 19.8)	19.5 (17.3 – 21.6)

Abbreviations: SNP = single nucleotide polymorphism; ECOG: Eastern Cooperative Oncology Group; PFS = progression free survival; OS = overall survival

Table S3. Hardy Weinberg Equilibrium *P*-values for the 21 genotyped SNPs in *VEGF-A, VEGFR1 and VEGFR2*

Gene	SNP	Hardy Weinberg *P*-value
VEGF-A	rs699946	0.0058
	rs699947	0.2615
	rs833061	0.0028
	rs1570360	1.0615×10^{-7}
	rs2010963	0.0888
	rs25648	0.9393
	rs3025039	0.0136
VEGFR1	rs9513070	9.4676×10^{-5}
	rs9554316	0.0068
	rs7993418	0.0033
	rs9582036	0.1275
	rs9554320	0.7055
VEGFR2	rs12505758	1
	rs7655964	0.465
	rs1870377	0.0091
	rs2305948	0.0529
	rs2305949	0.0208
	rs11133360	0.0729
	rs7667298	4.6002×10^{-8}
	rs2071559	2.0×10^{-4}
	rs1551641	0.0017

Abbreviations: SNP = single nucleotide polymorphism; *VEGF-A = vascular endothelial growth factor-A*; *VEGFR1 = vascular endothelial growth factor receptor 1*; *VEGFR2 = vascular endothelial growth factor receptor 2*

[a]Alleles **[1/2]** in the context sequences denote **[**VIC reporter-labelled Allele **1,** FAM reporter-labelled Allele **2]**

Table S4. Location and Minor Allele Frequency of 16 analysed SNPs

Gene	SNP	Location[a,b]	MAF
VEGF-A	rs699946	Distal Enhancer	0.14
	rs699947	Proximal Enhancer/Promoter[c]	0.47
	rs833061	Proximal Enhancer/Promoter[c]	0.48
	rs2010963	Promoter and 5’UTR/5’UTR[c]	0.31
	rs25648	Exon (synonymous substitution)/ 5’UTR[c]	0.13
	rs3025039	3’UTR	0.14
VEGFR1	rs9554316	Intron	0.19
	rs7993418	Exon (synonymous substitution)	0.20
	rs9582036	Intron	0.27
	rs9554320	Intron	0.41
VEGFR2	rs12505758	Intron	0.11
	rs7655964	Intron	0.32
	rs1870377	Exon- missense mutation	0.25
	rs2305948	Exon- missense mutation	0.10
	rs2305949	Intron	0.19
	rs11133360	Intron	0.43

[a]Unless otherwise stated, location data retrieved from the NCBI dbSNP Short Genetic Variations database (https://www.ncbi.nlm.nih.gov/snp), were mapped to GRCh38.p7 annotation release 108 using UCSC Genome Browser by referencing to the ENCODE Candidate Cis-Regulatory Elements (cCREs) track (Accession: EH38E2468791) - accessed 11th February 2021

[b]There are multiple locations listed for some SNPs - in these cases there are multiple transcripts, some of which have different locations

[c]Reference 37: Jain, L. *et al.* Hypertension and hand-foot skin reactions related to VEGFR2 genotype and improved clinical outcome following bevacizumab and sorafenib. *J Exp Clin Cancer Res* **29**, 95, doi:10.1186/1756-9966-29-95 (2010).

Abbreviations: SNP = single nucleotide polymorphism; *VEGF-A = vascular endothelial growth factor-A*; *VEGFR1 = vascular endothelial growth factor receptor 1*; *VEGFR2 = vascular endothelial growth factor receptor 2*; 5’UTR = 5’ untranslated region; 3’UTR – 3’ untranslated region; MAF = minor allele frequency

Table S5. Results of SNP genotyping for 16 SNPs in *VEGF-A, VEGFR1 and VEGFR2*

SNP information				Genotype (*n*, %)										
				Full genotype known				Partial genotype known			Undetermined genotype			
**Gene**	**SNP**	**Major allele**	**Minor allele**	**Homozygous major allele**	**Homozygous minor allele**	**Heterozygous major/ minor alleles**	***Total***	**Has major allele**	**Has minor allele**	***Total***	**Discordant genotyping calls**	**Failed to amplify**	**Insufficient template to perform genotyping**	***Total***
VEGF-A	rs699946	A	G	221 (68)	12 (4)	56 (17)	*289 (89)*	13 (4)	6 (2)	*19 (6)*	14 (4)	3 (1)	0 (0)	*17 (5)*
	rs699947	C	A	90 (28)	72 (22)	140 (43)	*302 (93)*	8 (2)	3 (1)	*11 (3)*	8 (2)	4 (1)	0 (0)	*12 (4)*
	rs833061	T	C	86 (26)	76 (23)	111 (34)	*273 (84)*	9 (3)	13 (4)	*22 (7)*	21 (6)	9 (3)	0 (0)	*30 (9)*
	rs2010963	G	C	150 (46)	35 (11)	114 (35)	*299 (92)*	11 (3)	6 (2)	*17 (5)*	7 (2)	2 (1)	0 (0)	*9 (3)*
	rs25648	C	T	236 (73)	4 (1)	69 (21)	*309 (95)*	4 (1)	5 (2)	*9 (3)*	6 (2)	1 (0.3)	0 (0)	*7 (2)*
	rs3025039	C	T	233 (72)	12 (4)	62 (19)	*307 (94)*	1 (0.3)	1 (0.3)	*2 (1)*	0 (0)	1 (0.3)	15 (5)	*16 (5)*
VEGFR1	rs9554316	G	T	209 (64)	19 (6)	78 (24)	*306 (94)*	5 (2)	12 (4)	*17 (5)*	2 (1)	0 (0)	0 (0)	*2 (1)*
	rs7993418	A	G	208 (64)	21 (6)	80 (25)	*309 (95)*	5 (2)	10 (3)	*15 (5)*	1 (0.3)	0 (0)	0 (0)	*1 (0.4)*
	rs9582036	A	C	170 (52)	29 (9)	112 (34)	*311 (96)*	6 (2)	4 (1)	*10 (3)*	2 (1)	2 (1)	0 (0)	*4 (1)*
	rs9554320	C	A	102 (31)	50 (15)	152 (47)	*304 (94)*	2 (1)	2 (1)	*4 (1)*	1 (0.3)	1 (0.3)	15 (5)	*17 (5)*
VEGFR2	rs12505758	T	C	242 (74)	4 (1)	61 (19)	*307 (94)*	2 (1)	10 (3)	*12 (4)*	4 (1)	2 (1)	0 (0)	*6 (2)*
	rs7655964	A	C	145 (45)	33 (10)	124 (38)	*302 (93)*	3 (1)	11 (3)	*14 (4)*	7 (2)	2 (1)	0 (0)	*9 (3)*
	rs1870377	T	A	184 (57)	29 (9)	99 (30)	*312 (96)*	1 (0.3)	5 (2)	*6 (2)*	4 (1)	3 (1)	0 (0)	*7 (2)*
	rs2305948	C	T	262 (81)	7 (2)	50 (15)	*319 (98)*	2 (1)	1 (0.3)	*3 (1)*	2 (1)	1 (0.3)	0 (0)	*3 (1)*
	rs2305949	C	T	204 (63)	18 (6)	80 (25)	*302 (93)*	11 (3)	2 (1)	*13 (4)*	8 (2)	2 (1)	0 (0)	*10 (3)*
	rs11133360	T	C	102 (31)	62 (19)	127 (39)	*291 (90)*	11 (3)	11 (3)	*22 (7)*	8 (2)	4 (1)	0 (0)	*12 (4)*

Abbreviations: SNP = single nucleotide polymorphism; *VEGF-A = vascular endothelial growth factor-A*; *VEGFR1 = vascular endothelial growth factor receptor 1*; *VEGFR2 = vascular endothelial growth factor receptor 2*

Table S6. Concordance in SNP genotype between fifty cases of matched colorectal normal and tumour tissue

Gene	SNP	Kendall's τ for concordance	*n*[a] used in analysis
VEGF-A	rs699946	1	40
	rs699947	0.94	43
	rs833061	0.84	38
	rs2010963	0.89	44
	rs25648	0.95	49
	rs3025039	0.95	47
VEGFR1	rs9554316	0.97	44
	rs7993418	0.91	46
	rs9582036	0.87	43
	rs9554320	0.86	48
VEGFR2	rs12505758	0.46	48
	rs7655964	0.85	46
	rs1870377	0.81	46
	rs2305948	0.91	47
	rs2305949	0.86	43
	rs11133360	0.79	42

Abbreviations: SNP = single nucleotide polymorphism; *VEGF-A = vascular endothelial growth factor-A*; *VEGFR1 = vascular endothelial growth factor receptor 1*; *VEGFR2 = vascular endothelial growth factor receptor*
[a]Full genotype known for both normal and tumour tissue samples

Table S7. Univariate associations between SNPs in *VEGF* family members and progression-free survival outcomes

Gene	SNP	Major allele	Minor allele	Homozygous major allele genotype versus without a homozygous major allele genotype					Homozygous minor allele genotype versus without a homozygous minor allele genotype				
				HR (95% CI)	*P*-value	Number of participants with homozygous major allele genotype	Number of participants without a homozygous major allele genotype	Total number of participants in analysis	HR (95% CI)	*P*-value	Number of participants with homozygous minor allele genotype	Number of participants without a homozygous minor allele genotype	Total number of participants in analysis
VEGF-A	rs699946	A	G	**1.40 (1.04 to 1.87)**	***P* = 0.025**	221	68	289	0.52 (0.27 to 1.00)	*P* = 0.050	12	277	289
	rs699947	C	A	0.78 (0.61 to 1.02)	*P* = 0.068	90	212	302	**1.32 (1.002 to 1.74)**	***P* = 0.048**	72	230	302
	rs833061	T	C	0.81 (0.62 to 1.05)	*P* = 0.12	86	187	273	1.26 (0.95 to 1.66)	*P* = 0.11	76	197	273
	rs2010963	G	C	1.14 (0.90 to 1.44)	*P* = 0.28	150	149	299	0.75 (0.51 to 1.09)	*P* = 0.13	35	264	299
	rs25648	C	T	**0.65 (0.49 to 0.85)**	***P* = 0.002**	236	73	309	0.67 (0.25 to 1.76)	*P* = 0.41	4	305	309
	rs3025039	C	T	0.84 (0.64 to 1.11)	*P* = 0.22	233	74	307	1.25 (0.67 to 2.33)	*P* = 0.49	12	295	307
VEGFR1	rs9554316	G	T	0.88 (0.69 to 1.14)	*P* = 0.34	209	97	306	0.97 (0.60 to 1.59)	*P* = 0.91	19	287	306
	rs7993418	A	G	0.91 (0.71 to 1.18)	*P* = 0.49	208	101	309	1.09 (0.68 to 1.76)	*P* = 0.71	21	288	309
	rs9582036	A	C	1.04 (0.82 to 1.31)	*P* = 0.76	170	141	311	1.12 (0.74 to 1.69)	*P* = 0.59	29	282	311
	rs9554320	C	A	0.86 (0.67 to 1.10)	*P* = 0.23	102	202	304	1.03 (0.74 to 1.42)	*P* = 0.87	50	254	304
VEGFR2	rs12505758	T	C	1.09 (0.82 to 1.45)	*P* = 0.54	242	65	307	0.41 (0.13 to 1.28)	*P* = 0.13	4	303	307
	rs7655964	A	C	0.79 (0.62 to 1.01)	*P* = 0.057	145	157	302	1.06 (0.72 to 1.57)	*P* = 0.76	33	269	302
	rs1870377	T	A	1.06 (0.84 to 1.35)	*P* = 0.61	184	128	312	0.96 (0.64 to 1.44)	*P* = 0.85	29	283	312
	rs2305948	C	T	0.76 (0.56 to 1.02)	*P* = 0.064	262	57	319	2.00 (0.88 to 4.53)	*P* = 0.099	7	312	319
	rs2305949	C	T	0.90 (0.70 to 1.16)	*P* = 0.41	204	98	302	0.78 (0.47 to 1.29)	*P* = 0.34	18	284	302
	rs11133360	T	C	0.86 (0.67 to 1.10)	*P* = 0.23	102	189	291	1.26 (0.94 to 1.69)	*P* = 0.13	62	229	291

Abbreviations: SNP = single nucleotide polymorphism; *VEGF-A = vascular endothelial growth factor-A*; *VEGFR1 = vascular endothelial growth factor receptor 1*; *VEGFR2 = vascular endothelial growth factor receptor 2*; HR = hazard ratio; 95% CI = 95% confidence interval

Table S8. Univariate associations between SNPs in *VEGF* family members and overall survival outcomes

Gene	SNP	Major allele	Minor allele	Homozygous major allele genotype versus without a homozygous major allele genotype					Homozygous minor allele genotype versus without a homozygous minor allele genotype				
				HR (95% CI)	*P*-value	Number of participants with homozygous major allele genotype	Number of participants without a homozygous major allele genotype	Total number of participants in analysis	HR (95% CI)	*P*-value	Number of participants with homozygous minor allele genotype	Number of participants without a homozygous minor allele genotype	Total number of participants in analysis
VEGF-A	rs699946	A	G	1.03 (0.75 to 1.41)	*P* = 0.85	221	68	289	1.02 (0.52 to 2.00)	*P* = 0.95	12	277	289
	rs699947	C	A	0.95 (0.71 to 1.27)	*P* = 0.73	90	212	302	1.14 (0.83 to 1.55)	*P* = 0.43	72	230	302
	rs833061	T	C	1.02 (0.75 to 1.37)	*P* = 0.93	86	187	273	1.06 (0.78 to 1.44)	*P* = 0.73	76	197	273
	rs2010963	G	C	0.85 (0.65 to 1.11)	*P* = 0.24	150	149	299	0.94 (0.62 to 1.43)	*P* = 0.76	35	264	299
	rs25648	C	T	**0.70 (0.52 to 0.94)**	***P* = 0.019**	236	73	309	0.46 (0.11 to 1.82)	*P* = 0.27	4	305	309
	rs3025039	C	T	1.06 (0.78 to 1.43)	*P* = 0.73	233	74	307	0.90 (0.43 to 1.88)	*P* = 0.78	12	295	307
VEGFR1	rs9554316	G	T	0.84 (0.64 to 1.11)	*P* = 0.21	209	97	306	1.10 (0.65 to 1.89)	*P* = 0.72	19	287	306
	rs7993418	A	G	0.85 (0.65 to 1.12)	*P* = 0.26	208	101	309	1.09 (0.65 to 1.83)	*P* = 0.74	21	288	309
	rs9582036	A	C	1.04 (0.80 to 1.35)	*P* = 0.79	170	141	311	0.91 (0.58 to 1.43)	*P* = 0.69	29	282	311
	rs9554320	C	A	0.77 (0.58 to 1.03)	*P* = 0.074	102	202	304	1.03 (0.72 to 1.48)	*P* = 0.86	50	254	304
VEGFR2	rs12505758	T	C	0.93 (0.67 to 1.28)	*P* = 0.63	242	65	307	0.55 (0.15 to 2.11)	*P* = 0.39	4	303	307
	rs7655964	A	C	0.88 (0.67 to 1.14)	*P* = 0.32	145	157	302	0.83 (0.54 to 1.28)	*P* = 0.40	33	269	302
	rs1870377	T	A	1.16 (0.89 to 1.51)	*P* = 0.28	184	128	312	0.83 (0.53 to 1.30)	*P* = 0.43	29	283	312
	rs2305948	C	T	0.84 (0.61 to 1.15)	*P* = 0.28	262	57	319	1.22 (0.53 to 2.83)	*P* = 0.64	7	312	319
	rs2305949	C	T	0.89 (0.67 to 1.17)	*P* = 0.41	204	98	302	0.78 (0.45 to 1.36)	*P* = 0.38	18	284	302
	rs11133360	T	C	0.91 (0.68 to 1.21)	*P* = 0.52	102	189	291	1.18 (0.85 to 1.63)	*P* = 0.33	62	229	291

Abbreviations: SNP = single nucleotide polymorphism; *VEGF-A = vascular endothelial growth factor-A*; *VEGFR1 = vascular endothelial growth factor receptor 1*; *VEGFR2 = vascular endothelial growth factor receptor 2*; HR = hazard ratio; 95% CI = 95% confidence interval

Table S9. Objective response rate by SNP genotypes in *VEGF-A, VEGFR1 and VEGFR2*

SNP information		Homozygous major allele genotype versus without a homozygous major allele genotype				Homozygous minor allele genotype versus without a homozygous minor allele genotype			
**Gene**	**SNP**								
VEGF-A	**rs699946**	**Treatment**	**AA genotype *n* (%)**	**GG and AG genotypes *n* (%)**	***P*-value[a]**	**Treatment**	**GG genotype *n* (%)**	**AA and AG genotypes *n* (%)**	***P*-value[a]**
		C	26 (79)	7 (21)	0.63	C	1(3)	32(97)	0.35
		CB	29 (78)	8 (22)		CB	3(8)	34(92)	
		CBM	31 (74)	11 (26)		CBM	1(2)	41(98)	
	**rs699947**	**Treatment**	**CC genotype *n* (%)**	**AA and CA genotypes *n* (%)**	***P*-value[a]**	**Treatment**	**AA genotype *n* (%)**	**CC and CA genotypes *n* (%)**	***P*-value[a]**
		C	12(34)	23(66)	0.43	C	9 (26)	26 (74)	0.70
		CB	17(45)	21(55)		CB	9 (24)	29 (76)	
		CBM	11(24)	34(76)		CBM	8 (18)	37 (82)	
	**rs833061**	**Treatment**	**TT genotype *n* (%)**	**CC and TC genotypes *n* (%)**	***P*-value[a]**	**Treatment**	**CC genotype *n* (%)**	**TT and TC genotypes *n* (%)**	***P*-value[a]**
		C	12(36)	21(64)	0.18	C	10 (30)	23 (70)	0.91
		CB	14(42)	19(58)		CB	9 (27)	24 (73)	
		CBM	10(26)	25(74)		CBM	9 (24)	29 (76)	
	**rs2010963**	**Treatment**	**GG genotype *n* (%)**	**CC and GC genotypes *n* (%)**	***P*-value[a]**	**Treatment**	**CC genotype *n* (%)**	**GG and GC genotypes *n* (%)**	***P*-value[a]**
		C	15(43)	20(57)	0.20	C	4 (11)	31 (89)	0.78
		CB	16(40)	24(60)		CB	7 (17)	33 (83)	
		CBM	24(56)	18(44)		CBM	4 (9)	39 (91)	
	**rs25648**	**Treatment**	**CC genotype *n* (%)**	**TT and CT genotypes *n* (%)**	***P*-value[a]**	**Treatment**	**TT genotype *n* (%)**	**CC and CT genotypes *n* (%)**	***P*-value[a]**
		C	29 (83)	6 (17)	0.77	C	0(0)	35(100)	0.31
		CB	29 (73)	11 (27)		CB	2(5)	38(95)	
		CBM	40 (87)	6 (13)		CBM	0(0)	46(100)	
	**rs3025039**	**Treatment**	**CC genotype *n* (%)**	**TT and CT genotypes *n* (%)**	***P*-value[a]**	**Treatment**	**TT genotype *n* (%)**	**CC and CT genotypes *n* (%)**	***P*-value[a]**
		C	31 (89)	4(11)	0.45	C	2(6)	33(94)	0.65
		CB	32(80)	8(80)		CB	2(5)	38(95)	
		CBM	32(70)	14(30)		CBM	1(2)	45(98)	

Table S9. Objective response rate by SNP genotypes in *VEGF-A, VEGFR1 and VEGFR2 continued*

SNP information		Homozygous major allele genotype versus without a homozygous major allele genotype				Homozygous minor allele genotype versus without a homozygous minor allele genotype			
**Gene**	**SNP**								
VEGF-R1	**rs9554316**	**Treatment**	**GG genotype *n* (%)**	**TT and GT genotypes *n* (%)**	***P*-value[a]**	**Treatment**	**TT genotype *n* (%)**	**GG and GT genotypes *n* (%)**	***P*-value[a]**
		C	21(71)	13(29)	0.061	C	2(6)	32(94)	0.69
		CB	28(72)	11(28)		CB	1(3)	38(97)	
		CBM	31(70)	13(30)		CBM	3(7)	41(93)	
	**rs7993418**	**Treatment**	**AA genotype *n* (%)**	**GG and AG genotypes *n* (%)**	***P*-value[a]**	**Treatment**	**GG genotype *n* (%)**	**AA and AG genotypes *n* (%)**	***P*-value[a]**
		C	21(60)	14(40)	0.56	C	3(9)	32(91)	0.46
		CB	27(69)	12(31)		CB	1(3)	38(97)	
		CBM	31(69)	14(31)		CBM	4(9)	41(91)	
	**rs9582036**	**Treatment**	**AA genotype *n* (%)**	**CC and AC genotypes *n* (%)**	***P*-value[a]**	**Treatment**	**CC genotype *n* (%)**	**AA and AC genotypes *n* (%)**	***P*-value[a]**
		C	18(53)	16(47)	0.59	C	5(15)	29(85)	0.87
		CB	23(57)	17(43)		CB	2(5)	38(95)	
		CBM	22(50)	22(50)		CBM	4(9)	40(91)	
	**rs9554320**	**Treatment**	**CC genotype *n* (%)**	**AA and CA genotypes *n* (%)**	***P*-value[a]**	**Treatment**	**AA genotype *n* (%)**	**CC and CA genotypes *n* (%)**	***P*-value[a]**
		C	13(38)	21(62)	0.50	C	6(18)	28(82)	0.53
		CB	17(43)	23(57)		CB	5(12)	35(88)	
		CBM	14(31)	31(69)		CBM	5(11)	40(89)	

Table S9. Objective response rate by SNP genotypes in *VEGF-A, VEGFR1 and VEGFR2 continued*

SNP information		Homozygous major allele genotype versus without a homozygous major allele genotype				Homozygous minor allele genotype versus without a homozygous minor allele genotype			
**Gene**	**SNP**								
VEGF-R2	**rs12502578**	**Treatment**	**TT genotype *n* (%)**	**CC and TC genotypes *n* (%)**	***P*-value[a]**	**Treatment**	**CC genotype *n* (%)**	**TT and TC genotypes *n* (%)**	***P*-value[a]**
		C	25(74)	9(26)	0.59	C	1(3)	33(97)	0.77
		CB	32(78)	9(22)		CB	1(2)	40(98)	
		CBM	33(80)	8(20)		CBM	0(0)	41(100)	
	**rs7655964**	**Treatment**	**AA genotype *n* (%)**	**CC and AC genotypes *n* (%)**	***P*-value[a]**	**Treatment**	**CC genotype *n* (%)**	**AA and AC genotypes *n* (%)**	***P*-value[a]**
		C	14(40)	21(60)	0.64	C	4(22)	31(89)	0.57
		CB	19(50)	19(50)		CB	2(5)	36(95)	
		CBM	23(56)	18(44)		CBM	2(5)	39(95)	
	**rs1870377**	**Treatment**	**TT genotype *n* (%)**	**AA and TA genotypes *n* (%)**	***P*-value[a]**	**Treatment**	**AA genotype *n* (%)**	**TT and TA genotypes *n* (%)**	***P*-value[a]**
		C	18(53)	16(47)	0.35	C	2(6)	32(94)	0.54
		CB	24(59)	17(41)		CB	7(17)	34(83)	
		CBM	25(57)	19(43)		CBM	3(7)	41(93)	
	**rs2305948**	**Treatment**	**CC genotype *n* (%)**	**TT and CT genotypes *n* (%)**	***P*-value[a]**	**Treatment**	**TT genotype *n* (%)**	**CC and CT genotypes *n* (%)**	***P*-value[a]**
		C	28(78)	8(22)	0.51	C	1(3)	35(97)	0.37
		CB	36(86)	6(14)		CB	0(0)	42(100)	
		CBM	33(75)	11(25)		CBM	2(2)	43(98)	
	**rs2305949**	**Treatment**	**CC genotype *n* (%)**	**TT and CT genotypes *n* (%)**	***P*-value[a]**	**Treatment**	**TT genotype *n* (%)**	**CC and CT genotypes *n* (%)**	***P*-value[a]**
		C	22(67)	11(33)	0.53	C	1(3)	32(97)	0.31
		CB	24(69)	11(31)		CB	1(3)	34(97)	
		CBM	23(53)	20(47)		CBM	4(9)	39(91)	
	**rs11133360**	**Treatment**	**TT genotype *n* (%)**	**CC and TC genotypes *n* (%)**	***P*-value[a]**	**Treatment**	**CC genotype *n* (%)**	**TT and TC genotypes *n* (%)**	***P*-value[a]**
		C	16(47)	18(53)	0.85	C	5(15)	28(85)	0.66
		CB	12(34)	23(66)		CB	7(20)	28(80)	
		CBM	14(33)	28(67)		CBM	7(17)	35(83)	

Abbreviations: *VEGF-A = vascular endothelial growth factor-A*; *VEGF-R1 = vascular endothelial growth factor receptor 1*; *VEGF-R2 = vascular endothelial growth factor receptor 2*; C= capecitabine; CB = capecitabine and bevacizumab; CBM = capecitabine, bevacizumab and mitomycin

[a]*P*-value for interaction between SNP genotype biomarker status and the allocated treatment (‘CB + CBM’ *vs* ‘C’)

Table S10. Grade ≥ 3 hypertension by SNP genotypes in *VEGF-A, VEGFR1 and VEGFR2*

Gene	SNP	Major allele	Minor allele	Homozygous major allele genotype versus without a homozygous major allele genotype					Homozygous minor allele genotype versus without a homozygous minor allele genotype				
				OR (95% CI)	*P*-value	Number of participants with homozygous major allele genotype	Number of participants without a homozygous major allele genotype	Total number of participants in analysis	OR (95% CI)	*P*-value	Number of participants with homozygous minor allele genotype	Number of participants without a homozygous minor allele genotype	Total number of participants in analysis
VEGF-A	rs699946	A	G	**A** 0.71 (0.18 – 2.82)<br>**B** 0.69 (0.17 – 2.81)	*P* = 0.63<br>*P* = 0.61	221	68	289	**A** not estimable[a]<br>**B** not estimable[a]	*P* = 0.64[b]	12	277	289
	rs699947	C	A	**A** 0.51 (0.11 – 2.42)<br>**B** 0.53 (0.11 – 2.51)	*P* = 0.40<br>*P* = 0.42	90	212	302	**A** 0.70 (0.15 – 3.32)<br>**B** 0.76 (0.16 – 3.67)	*P* = 0.66<br>*P* = 0.74	72	230	302
	rs833061	T	C	**A** 0.61 (0.13 – 3.01)<br>**B** 0.61 (0.12 – 2.99)	*P* = 0.55<br>*P* = 0.54	86	187	273	**A** 0.73 (0.15 – 3.61)<br>**B** 0.78 (0.16 – 3.86)	*P* = 0.70<br>*P* = 0.76	76	197	273
	rs2010963	G	C	**A** 0.49 (0.12 – 1.98)<br>**B** 0.53 (0.13 – 2.17)	*P* = 0.32<br>*P* = 0.38	150	149	299	**A** not estimable[a]<br>**B** not estimable[a]	*P* = 0.25[b]	35	264	299
	rs25648	C	T	**A** not estimable[a]<br>**B** not estimable[a]	*P* = 0.06[b]	236	73	309	**A** not estimable[a]<br>**B** not estimable[a]	*P* = 0.70[b]	4	305	309
	rs3025039	C	T	**A** 0.54 (0.15 – 1.91)<br>**B** 0.60 (0.17 – 2.15)	*P* = 0.34<br>*P* = 0.44	233	74	307	**A** 2.58 (0.30 – 22.04)<br>**B** 2.65 (0.30 – 23.53)	*P* = 0.39<br>*P* = 0.38	12	295	307
VEGFR1	rs9554316	G	T	**A** 2.14 (0.45 – 10.07)<br>**B** 2.05 (0.43 – 9.75)	*P* = 0.34<br>*P* = 0.37	209	97	306	**A** not estimable[a]<br>**B** not estimable[a]	*P* = 0.39[b]	19	287	306
	rs7993418	A	G	**A** 2.24 (0.47 – 10.54)<br>**B** 2.20 (0.46– 10.45)	*P* = 0.31<br>*P* = 0.32	208	101	309	**A** not estimable[a]<br>**B** not estimable[a]	*P* = 0.38[b]	21	288	309
	rs9582036	A	C	**A** 1.47 (0.42 – 5.13)<br>**B** 1.40 (0.40 – 4.92)	*P* = 0.55<br>*P* = 0.60	170	141	311	**A** 0.98 (0.12 – 7.90)<br>**B** 1.12 (0.14 – 9.28)	*P* = 0.99<br>*P* = 0.92	29	282	311
	rs9554320	C	A	**A** 2.03 (0.57 – 7.18)<br>**B** 2.07 (0.58 – 7.42)	*P* = 0.27<br>*P* = 0.27	102	202	304	**A** 0.56 (0.07 – 4.49)<br>**B** 0.62 (0.08 – 5.05)	*P* = 0.58<br>*P* = 0.65	50	254	304
VEGFR2	rs12505758	T	C	**A** 2.76 (0.35 – 21.94)<br>**B** 2.77 (0.34 – 22.21)	*P* = 0.34<br>*P* = 0.34	242	65	307	**A** not estimable[a]<br>**B** not estimable[a]	*P* = 0.59[b]	4	303	307
	rs7655964	A	C	**A** 0.90 (0.27 – 3.01)<br>**B** 0.84 (0.25 – 2.84)	*P* = 0.86<br>*P* = 0.77	145	157	302	**A** not estimable[a]<br>**B** not estimable[a]	*P* = 0.24[b]	33	269	302
	rs1870377	T	A	**A** 0.38 (0.11 – 1.34)<br>**B** 0.40 (0.11 – 1.42)	*P* = 0.13<br>*P* = 0.16	184	128	312	**A** 0.99 (0.12 – 7.92)<br>**B** 0.89 (0.11 – 7.30)	*P* = 0.99<br>*P* = 0.91	29	283	312
	rs2305948	C	T	**A** 2.22 (0.28 – 17.71)	*P* = 0.45	262	57	319	**A** not estimable[a]	*P* = 0.62[b]	7	312	319
				**B** 2.36 (0.29 – 19.09)	*P* = 0.42				**B** not estimable[a]				
	rs2305949	C	T	**A** 1.96 (0.41 – 9.39)	*P* = 0.40	204	98	302	**A** not estimable[a]	*P* = 0.40[b]	18	284	302
				**B** 2.21 (0.45 – 10.75)	*P* = 0.33				**B** not estimable[a]				
	rs11133360	T	C	**A** not estimable[a]	***P* = 0.03[b]**	102	198	291	**A** 1.03 (0.21 – 5.13)	*P* = 0.97	62	229	291
				**B** not estimable[a]					**B** 1.17 (0.23 – 5.90)	*P* = 0.85			

Abbreviations: SNP = single nucleotide polymorphism; *VEGF-A = vascular endothelial growth factor-A*; *VEGFR1 = vascular endothelial growth factor receptor 1*; *VEGFR2 = vascular endothelial growth factor receptor 2*; OR = odds ratio; 95% CI = 95% confidence interval

A: analysis with SNP genotype alone; B: analysis with both the SNP genotype and allocated treatment covariates (‘CB and CBM’ versus ‘C’); [a]: OR not estimable due to zero events in one cell of the 2x2 contingency table; [b]: Conditional Binomial Exact Test (CBET) for independence between SNP and hypertension.

Figure S1. Characteristics of tissue used for SNP genotyping

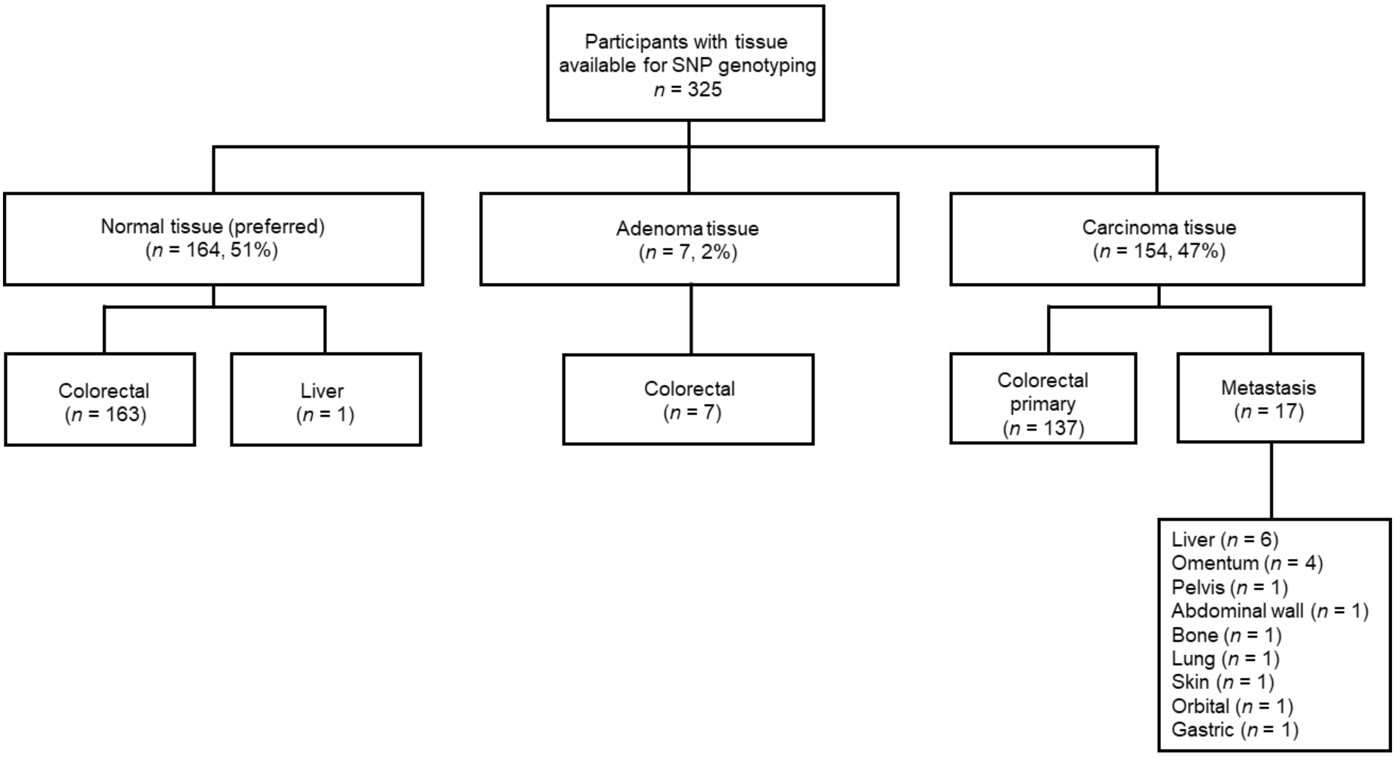

Figure S1 legend: Flow diagram showing the histopathological diagnosis and location of tissue used for SNP genotyping in the MAX VEGF SNP sub-study**.** Abbreviation: SNP = single nucleotide polymorphism

Figure S2. Genomic location of *VEGFR2* rs11133360 and predicted functional effects on *VEGFR*2 expression

a

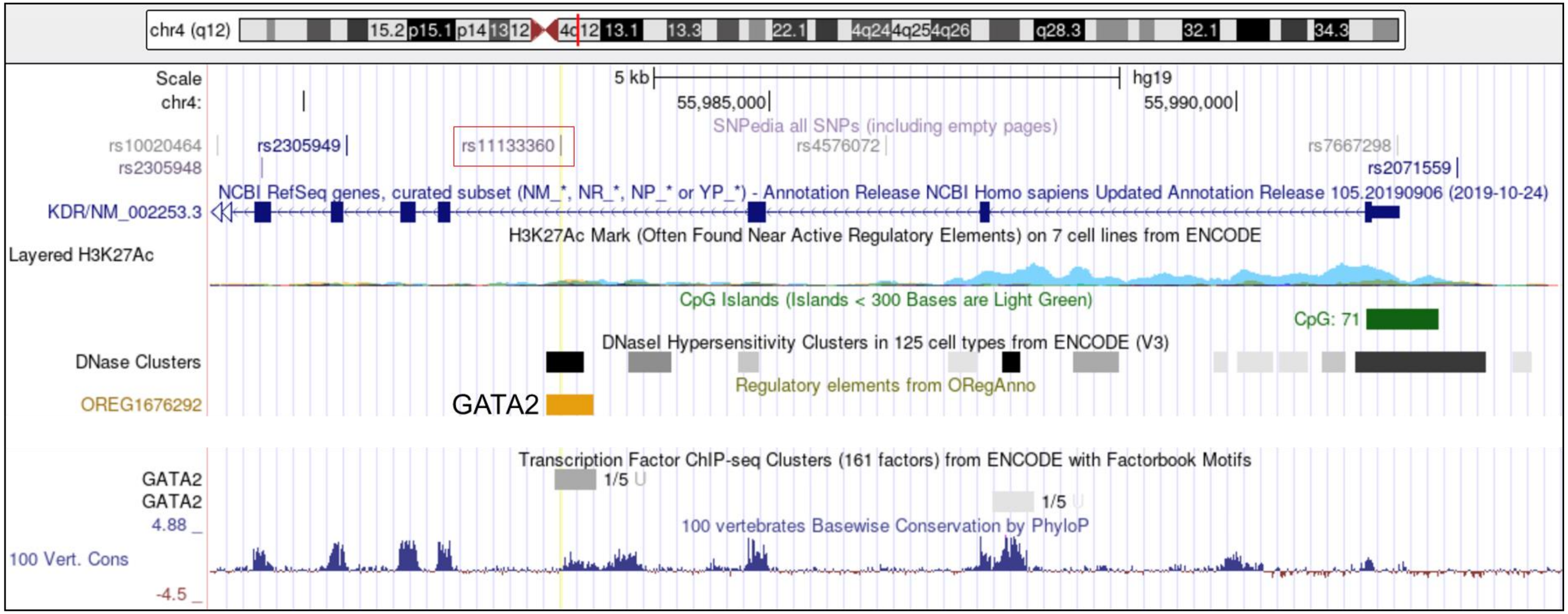

b

GATA2 binding consensus sequence: 5'-(A/T)GATA(A/G)-3' *VEGFR2* rs11133360 shaded in yellow

TT genotype – wild-type GATA2 binding site
Forward Sequence: CTTGATAGGA
Reverse Sequence: GAACTATCCT

CC genotype – mutant GATA2 binding site (loss of GATA2 binding)
Forward Sequence: CTTGATGGGA
Reverse Sequence: GAACTACCCT

Figure S2. Genomic location of *VEGFR2* rs11133360 and predicted functional effects on *VEGFR*2 expression *continued*

Figure S2 legend: Genomic location of *VEGFR2* rs11133360. Location data retrieved from the NCBI dbSNP Short Genetic Variations database (https://www.ncbi.nlm.nih.gov/snp) was mapped to GRCh38.p7 annotation release 108 using the UCSC Genome Browser by referencing to multiple Gene Regulatory Element databases, including the ENCODE Candidate Cis-Regulatory Elements (cCREs) track (Accession: EH38E2468791), ORegAnno database[1] and Transcription Factor ChIP-seq Clusters from ENCODE with Factorbook Motifs[2]. **(a)** *VEGFR2* rs11133360 is located in the third intron of the human *VEGFR2* gene, ~7 kb from the promoter and 5' UTR. This SNP lies within a GATA2 binding region (ID: OREG1676292), as annotated in the ORegAnno database. Importantly, this GATA2 binding region was independently verified using ChIP-seq analysis with an anti-GATA2 antibody in human umbilical vein endothelial cells (HUVECs) by the ENCODE consortium. It also coincides with a strong DNAase I hypersensitivity region (i.e. indicative of an open chromatin structure that commonly associates with transcription factor binding regions at promoter or enhancer regions). Of note, ~ 3 kb upstream of the rs11133360-associated GATA2 binding region, in the first intron of the *VEGFR2* gene, there is an additional GATA2 binding region (identified by the ENCODE ChIP-seq dataset), which is also associated with a strong DNAase I hypersensitivity region. Sequence alignment analysis indicated that this second GATA2 binding region forms part of the experimentally verified enhancer sequence that is critical for the expression of mouse isoforms of *VEGFR2* (Flk-1) *in vivo*[3]. Therefore, it is likely that the rs11133360-associated GATA2 binding region can also function to regulate *VEGFR2* expression in humans. **(b)** Bioinformatic analysis using TFBIND (http://tfbind.hgc.jp/) confirmed that *VEGFR2* rs11133360 mapped to a putative GATA2 binding site identified based on the consensus *in vitro* GATA2 binding sequence. A TT genotype encompasses a wild-type GATA2 binding sequence. In contrast, a CC genotype results in a mutated GATA2 binding site, which is likely to result in the loss of GATA2 binding. Together, this analysis provides key supporting evidence that a non-TT genotype is likely to be associated with reduced *VEGFR2* expression due to the loss of a functional GATA2 binding site, as GATA2 has previously been demonstrated to be a bona fide transcription factor that regulates the expression of mouse isoforms of *VEGFR2* (Flk-1)[3].

References

1. Lesurf, R. *et al.* Open Regulatory Annotation Consortium. ORegAnno 3.0: a community-driven resource for curated regulatory annotation. *Nucleic Acids Res.* **44(D1),** D126-32. doi: 10.1093/nar/gkv1203 (2016).
2. Wang, J. *et al.* Factorbook.org: a Wiki-based database for transcription factor-binding data generated by the ENCODE consortium. *Nucleic Acids Res.* **41(Database issue),** D171-6. doi: 10.1093/nar/gks1221 (2013).
3. Kappel, A. *et al.* Role of SCL/Tal-1, GATA, and ets transcription factor binding sites for the regulation of flk-1 expression during murine vascular development. *Blood.* **96**, 3078-85 (2000).
